# Meta‐analysis of first‐line therapies with maintenance regimens for advanced non‐small‐cell lung cancer (NSCLC) in molecularly and clinically selected populations

**DOI:** 10.1002/cam4.1101

**Published:** 2017-07-03

**Authors:** Pui San Tan, Marcel Bilger, Gilberto de Lima Lopes, Sanchalika Acharyya, Benjamin Haaland

**Affiliations:** ^1^ Nuffield Department of Primary Care Health Sciences University of Oxford Oxford United Kingdom; ^2^ Health Services & Systems Research Duke‐NUS Graduate Medical School Singapore Singapore; ^3^ Sylvester Comprehensive Cancer Center University of Miami and the Miller School of Medicine Miami Florida; ^4^ Centre for Quantitative Medicine Duke‐NUS Graduate Medical School Singapore Singapore; ^5^ H. Milton Stewart School of Industrial and Systems Engineering Georgia Institute of Technology Atlanta Georgia

**Keywords:** advanced non‐small‐cell lung cancer (NSCLC), Bayesian network meta‐analysis, first‐line with maintenance therapy, molecularly and clinically selected patients

## Abstract

Evidence has suggested survival benefits of maintenance for advanced NSCLC patients not progressing after first‐line chemotherapy. Additionally, particular first‐line targeted therapies have shown survival improvements in selected populations. Optimal first‐line and maintenance therapies remain unclear. Here, currently available evidence was synthesized to elucidate optimal first‐line and maintenance therapy within patient groups. Literature was searched for randomized trials evaluating first‐line and maintenance regimens in advanced NSCLC patients. Bayesian network meta‐analysis was performed within molecularly and clinically selected groups. The primary outcome was *combined clinically meaningful OS and PFS benefits*. A total of 87 records on 56 trials evaluating first‐line treatments with maintenance were included. Results showed combined clinically meaningful OS and PFS benefits with particular first‐line with maintenance treatments, (1) first‐line intercalated chemotherapy+erlotinib, maintenance erlotinib in patients with EGFR mutations, (2) first‐line afatinib, maintenance afatinib in patients with EGFR deletion 19, (3) first‐line chemotherapy + bevacizumab, maintenance bevacizumab in EGFR wild‐type patients, (4) chemotherapy+conatumumab, maintenance conatumumab in patients with squamous histology, (5) chemotherapy+cetuximab, maintenance cetuximab or chemotherapy + necitumumab, maintenance necitumumab in EGFR FISH‐positive patients with squamous histology, and (6) first‐line chemotherapy+bevacizumab, maintenance bevacizumab or first‐line sequential chemotherapy+gefitinib, maintenance gefitinib in patients clinically enriched for EGFR mutations with nonsquamous histology. No treatment showed combined clinically meaningful OS and PFS benefits in patients with EGFR L858R or nonsquamous histology. Particular first‐line with maintenance treatments show meaningful OS and PFS benefits in patients selected by EGFR mutation or histology. Further research is needed to achieve effective therapy for patients with EGFR mutation L858R or nonsquamous histology.

## Introduction

Lung cancer remains the most common cause of cancer deaths in men 1, with only 30% surviving beyond 1 year and 8% beyond 5 years 2. Conventional first‐line therapy for patients with advanced NSCLC has been four to six cycles of chemotherapy doublets 3. On the other hand, emerging evidence has shown potential benefits of administering maintenance therapies to nonprogressing patients beyond four to six cycles of chemotherapy until disease progression 4. However, survival benefits have only been observed for particular maintenance treatments in targeted patient populations, for example, switch to or continue pemetrexed in patients with nonsquamous histology or continue gemcitabine in patients with squamous histology 4. Basic research has corroborated these clinical findings by showing that administration of effective therapies before disease progression could enhance kill of tumor cells before onset of treatment resistance 5, 6.

Optimal *combined* first‐line with maintenance treatment remains unknown. In fact, a sizeable portion of evidence on maintenance benefits has been derived from trials that randomized treatments only to patients not progressing after first‐line chemotherapy, and subsequently measured survival and progression times from start of maintenance therapy 7, 8, 9, 10. Many other trials have, in contrast, randomized patients at onset of first‐line therapy, and subsequently measured survival and progression times from start of first‐line therapy, then *through* maintenance 11, 12, 13. Moreover, many maintenance trials have evaluated maintenance after first‐line *chemotherapy*, while emerging evidence has shown benefits of first‐line targeted therapies compared to standard first‐line chemotherapy in particular patient populations 14. With that, there remains a paucity of evidence for optimal combination or sequence of first‐line and maintenance regimens.

Additionally, key trials in the first‐line setting have shown little or no survival benefit of first‐line therapies with maintenance regimens in unselected populations 7, 8, 9, 10. Nevertheless, survival benefits have been observed when patients are selected by particular biomarkers 14. For example afatinib has demonstrated survival benefits in patients harboring EGFR mutation subtype deletion 19 14. Survival benefits have been suggested within other clinically or molecularly selected populations 15. For example, patients with squamous histology and EGFR FISH positive showed survival gains with first‐line chemotherapy and necitumumab maintenance 15.

Furthermore, the majority of first‐line trials with maintenance regimens have been compared to standard chemotherapy with no maintenance 11, 12, rendering a lack of reliable evidence on head‐to‐head comparisons of treatments. With emerging evidence of survival benefits in targeted populations 14, elucidating treatment strategies for clinically and molecularly selected patients is essential.

In order to elucidate first‐line and maintenance treatments that would have the most benefit for patients, one must consider outcomes that are *clinically meaningful* for patients. In lung cancer, an improvement of 3–4 months of survival or an HR around 0.80 might be considered clinically meaningful 16.

In this study, first‐line treatments with maintenance regimens are compared head‐to‐head via network meta‐analysis in terms of combined clinically meaningful overall survival (OS) and progression‐free survival (PFS) benefits. Treatments are compared from a precision medicine perspective in terms of treatment benefits within (1) molecularly selected patients in terms of EGFR mutation positive versus wild type, EGFR mutation subtype deletion 19 versus L858R, EGFR FISH, and (2) clinically selected patients in terms of histology and clinically enriched EGFR populations.

## Methods

### Systematic review and study selection

#### Search strategy

**Figure 1:**
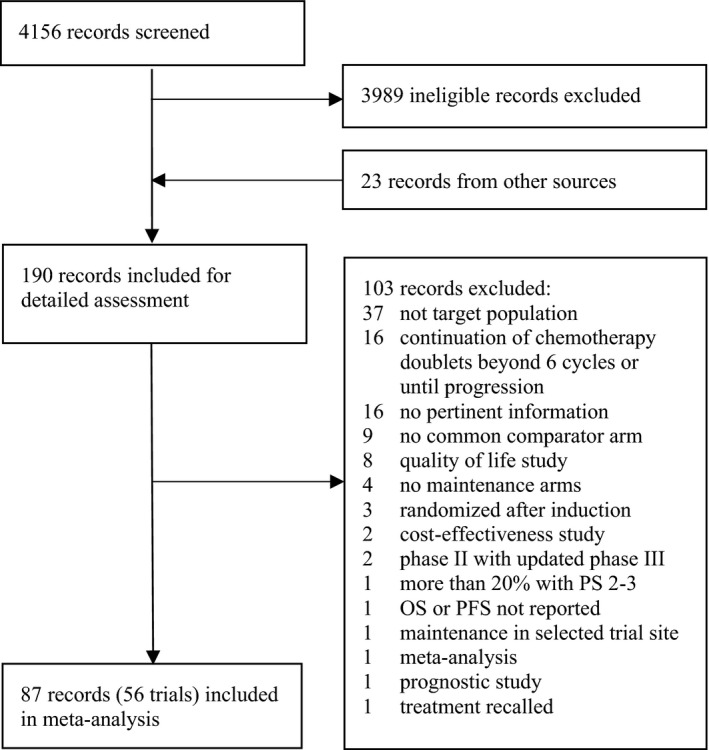
Search diagram for trials evaluating first‐line therapies followed by maintenance regimens in advanced NSCLC patients according to PRISMA 37 guidelines.

PubMed was searched for relevant studies published from 1 December 2003 to 19 March 2015. Phase II/III randomized controlled trials evaluating first‐line treatments with maintenance regimens in advanced NSCLC patients reporting OS or PFS relative efficacy estimates were included. Conference proceedings (ASCO 2014‐2015, ESMO 2014) were searched for additional relevant studies 17, 18. Studies which (1) randomized patients after first‐line treatment, (2) continued chemotherapy doublets beyond six cycles or until progression, (3) included more than 20% patients with performance (PS) 2–3, or (4) included surgery, radiation, or chemoradiation as treatment arms were excluded. Detailed accounting of studies is provided in Figure 1. Study screening was performed by two independent reviewers and disagreements were discussed with the team until consensus. Individual trial characteristics and relative efficacy estimates for OS and PFS were extracted from the included studies.

#### Outcomes evaluation

Combined clinically meaningful OS and PFS benefits was defined as hazard ratios (HRs) ≤0.80 16 with ≥95% posterior probability of the treatment being better than standard chemotherapy with no maintenance. Treatment efficacies were evaluated in terms of (1) surface under the cumulative ranking curve (SUCRA) 19, (2) posterior HR with corresponding 95% credible interval (CrI), (3) posterior probability better than standard chemotherapy with no maintenance, and (4) posterior probability the treatment is best. SUCRAs were computed as the average cumulative probabilities for a particular treatment to be ranked best, top two, top three, and so on 19.

Treatment efficacies were meta‐analyzed within the following molecularly and clinically selected subgroups, (1) EGFR mutation positive, (2) EGFR mutation subtype deletion 19, (3) EGFR mutation subtype L858R, (4) EGFR wild type, (5) EGFR FISH positive, (6) nonsquamous histology, (7) squamous histology, and (8) clinically enriched for EGFR mutation. The clinically enriched for EGFR mutation population was defined as patients of Asian/East‐Asian origins or who were light or never smokers 20. Additional head‐to‐head comparisons of treatments for EGFR mutation/subtypes and wild type were performed.

### Statistical analysis

Bayesian network meta‐analysis (NMA) was performed by separately pooling individual studies’ reported OS and PFS hazard ratios on the logarithmic scale. Log hazard ratios were modeled as normally distributed centered on a treatment contrast‐specific mean subject to within‐ and between‐study heterogeneities. For studies which did not fully report HRs and confidence intervals (CIs), efficacies were computed using procedures outlined in Tierney et al. 21.

Prior distributions for within‐study heterogeneities were inverse gamma with mean matching the corresponding study's reported variance and variance proportional to the number of events reported for each endpoint in each study. Prior distributions for average treatment efficacies (log hazard ratios) were modeled uninformatively as normal centered at zero with large variance. Between‐study heterogeneity priors were weakly informative uniforms placing 95% of the prior mass on relative treatment efficacies varying up to twofold between studies.

Draws from the posterior distribution were generated using 10 Markov chain Monte Carlo chains, each with 100,000 burn‐in simulations followed by posterior sampling of 100,000 observations each to generate posterior efficacies in terms of treatment HRs and respective 95% CrIs, SUCRA rankings, probability best, and probability better than standard chemotherapy with no maintenance.

Multiple efficacy estimates from the same study were modeled as multivariate normal with a study correlation. A uniform prior on 0 to 0.95 was specified for the within‐study correlation. Bayesian meta‐analysis was implemented using JAGS 22 run via the R interface rjags 23. All other statistical analyses were performed in R 24. WebPlotDigitizer was used to recover HRs and respective CIs reported graphically in individual studies 25.

## Results

**Figure 2:**
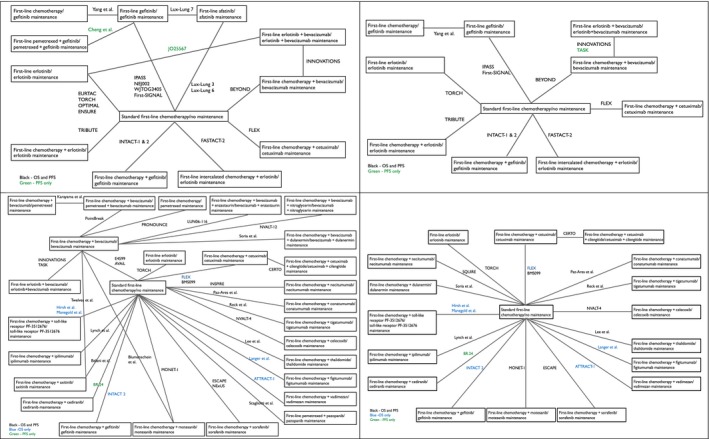
Network of studies in (A) EGFR mutation positive (top left), (B) EGFR wild type (top right), (C) nonsquamous (bottom left), and (D) squamous (bottom right).

A total of 87 records and 56 trials evaluating first‐line with maintenance treatments in advanced NSCLC were included for meta‐analysis (Fig. 1). Studies and treatment comparisons within‐patient groups are shown in Figure 2 and Appendix Table 1.

### Molecularly selected populations

#### EGFR mutation

##### EGFR mutation positive

**Figure 3:**
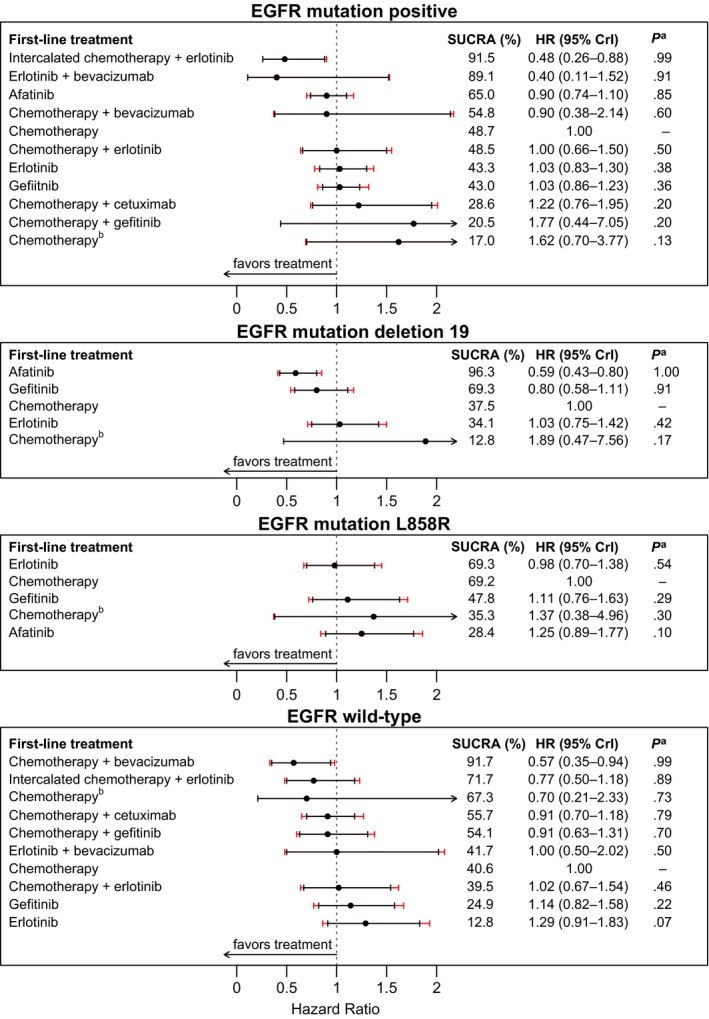
Overall survival hazard ratio (95% CrI), surface under the cumulative ranking curve (SUCRA) probability, and probability^a^ better than standard chemotherapy with no maintenance by EGFR mutation status and subtypes for first‐line therapies with corresponding maintenance regimens. For intercalated chemotherapy+erlotinib, erlotinib was administered as 150 mg daily days 15–28 every 28 days cycle 28. ^a^Posterior probability better than standard chemotherapy with no maintenance. ^b^First‐line chemotherapy followed by gefitinib maintenance.

In EGFR mutation‐positive patients, first‐line intercalated chemotherapy + erlotinib with erlotinib maintenance was the only treatment which showed combined clinically meaningful OS and PFS benefits. First‐line intercalated chemotherapy + erlotinib with erlotinib maintenance showed the best survival SUCRA, along with posterior HR 0.48 (0.26–0.88) and 99% posterior probability of outperforming chemotherapy with no maintenance (Table 1 and Fig. 3). Clinically meaningful PFS benefits were demonstrated with first‐line intercalated chemotherapy + erlotinib, erlotinib + bevacizumab, afatinib, chemotherapy + bevacizumab, erlotinib, gefitinib, chemotherapy (gefitinib maintenance), and pemetrexed + gefitinib, each with maintenance regimens as illustrated in Table 1 and Appendix Figure 4.

**Table 1:** Overall survival and progression‐free survival by EGFR mutation status and subtypes

First‐line treatment	Maintenance treatment	Overall survival				Progression‐free survival		
		SUCRA	HR (95% CrI)	Probability better than standard chemotherapy with no maintenance	Probability best	HR (95% CrI)	Probability better than standard chemotherapy with no maintenance	Probability best
EGFR mutation positive								
Intercalated chemotherapy+erlotinib^b^	Erlotinib^b^	91.5%^a^	0.48 (0.26–0.88)	0.99	0.39	0.25 (0.15–0.43)	1.00	0.13
Erlotinib+bevacizumab	Erlotinib+bevacizumab	89.1%	0.40 (0.11–1.52)	0.91	0.58	0.18 (0.11–0.30)	1.00	0.57
Afatinib	Afatinib	65.0%	0.90 (0.74–1.10)	0.85	0.00	0.38 (0.29–0.49)	1.00	0.00
Chemotherapy+bevacizumab	Bevacizumab	54.8%	0.90 (0.38–2.14)	0.60	0.01	0.23 (0.12–0.45)	1.00	0.18
Chemotherapy	No maintenance	48.7%	1.00	–	0.00	1.00	–	0.00
Chemotherapy+erlotinib	Erlotinib	48.5%	1.00 (0.66–1.50)	0.50	0.00	0.49 (0.18–1.31)	0.93	0.02
Erlotinib	Erlotinib	43.3%	1.03 (0.83–1.30)	0.38	0.00	0.32 (0.24–0.42)	1.00	0.00
Gefitinib	Gefitinib	43.0%	1.03 (0.86–1.23)	0.36	0.00	0.45 (0.37–0.56)	1.00	0.00
Chemotherapy+cetuximab	Cetuximab	28.6%	1.22 (0.76–1.95)	0.20	0.00	0.70 (0.43–1.14)	0.93	0.00
Chemotherapy+gefitinib	Gefitinib	20.5%	1.77 (0.44–7.05)	0.20	0.02	0.55 (0.17–1.82)	0.85	0.03
Chemotherapy	Gefitinib	17.0%	1.62 (0.70–3.77)	0.13	0.00	0.38 (0.17–0.82)	0.99	0.03
Pemetrexed+gefitinib	Pemetrexed+gefitinib	–	–	–	–	0.31 (0.19–0.51)	1.00	0.03
EGFR mutation Del 19								
Afatinib	Afatinib	96.3%^a^	0.59 (0.43–0.80)	1.00	0.87	0.24 (0.18–0.32)	1.00	0.00
Gefitinib	Gefitinib	69.3%	0.80 (0.58–1.11)	0.91	0.08	0.33 (0.26–0.43)	1.00	0.00
Chemotherapy	No maintenance	37.5%	1.00	–	0.00	1.00	–	0.00
Erlotinib	Erlotinib	34.1%	1.03 (0.75–1.42)	0.42	0.00	0.20 (0.14–0.29)	1.00	0.00
Chemotherapy	Gefitinib	12.8%	1.89 (0.47–7.56)	0.17	0.05	–	–	–
Erlotinib+bevacizumab	Erlotinib+bevacizumab	–	–	–	–	0.08 (0.04–0.17)	1.00	1.00
EGFR mutation L858R								
Erlotinib	Erlotinib	69.3%	0.98 (0.70–1.38)	0.54	0.37	0.43 (0.29–0.63)	1.00	0.06
Chemotherapy	No maintenance	69.2%	1.00	–	0.22	1.00	–	0.00
Gefitinib	Gefitinib	47.8%	1.11 (0.76–1.63)	0.29	0.12	0.55 (0.42–0.72)	1.00	0.01
Chemotherapy	Gefitinib	35.3%	1.37 (0.38–4.96)	0.30	0.25	–	–	–
Afatinib	Afatinib	28.4%	1.25 (0.89–1.77)	0.10	0.04	0.45 (0.33–0.62)	1.00	0.11
Erlotinib+bevacizumab	Erlotinib+bevacizumab	–	–	–	–	0.29 (0.14–0.60)	1.00	0.82
EGFR wild type								
Chemotherapy+bevacizumab	Bevacizumab	91.7%^a^	0.57 (0.35–0.94)	0.99	0.52	0.33 (0.20–0.55)	1.00	0.64
Intercalated chemotherapy+erlotinib	Erlotinib^b^	71.7%	0.77 (0.50–1.18)	0.89	0.09	0.97 (0.65–1.45)	0.56	0.00
Chemotherapy	Gefitinib	67.3%	0.70 (0.21–2.33)	0.73	0.34	0.42 (0.12–1.40)	0.93	0.36
Chemotherapy+cetuximab	Cetuximab	55.7%	0.91 (0.70–1.18)	0.79	0.01	1.02 (0.78–1.34)	0.43	0.00
Chemotherapy+gefitinib	Gefitinib	54.1%	0.91 (0.63–1.31)	0.70	0.02	0.73 (0.49–1.08)	0.95	0.00
Erlotinib+bevacizumab	Erlotinib+bevacizumab	41.7%	1.00 (0.50–2.02)	0.50	0.01	0.68 (0.36–1.29)	0.88	0.00
Chemotherapy	No maintenance	40.6%	1.00	–	0.00	1.00	–	0.00
Chemotherapy+erlotinib	Erlotinib	39.5%	1.02 (0.67–1.54)	0.46	0.01	1.24 (0.86–1.79)	0.12	0.00
Gefitinib	Gefitinib	24.9%	1.14 (0.82–1.58)	0.22	0.00	2.32 (1.65–3.24)	0.00	0.00
Erlotinib	Erlotinib	12.8%	1.29 (0.91–1.83)	0.07	0.00	2.07 (1.46–2.92)	0.00	0.00

a Treatments showing clinically meaningful benefits defined as HR ≤0.8 and probability better than standard chemotherapy with no maintenance ≥0.95 for both OS and PFS. HR, hazard ratio; CrI, credible intervals.

b For intercalated chemotherapy+erlotinib, erlotinib was administered as 150 mg daily days 15–28 every 28 days cycle [28].

Head‐to‐head comparisons for OS showed that first‐line intercalated chemotherapy + erlotinib with erlotinib maintenance outperformed first‐line chemotherapy + erlotinib, erlotinib, gefitinib, chemotherapy + cetuximab, and chemotherapy (gefitinib maintenance), each with maintenance regimens as illustrated in Appendix Table 17. Head‐to‐head comparisons for PFS benefits are shown in Appendix Table 18.

##### EGFR mutation subtype Del 19

In patients with EGFR mutation subtype Del 19, first‐line afatinib with maintenance afatinib was the only treatment which showed combined clinically meaningful OS and PFS benefits. First‐line afatinib with maintenance afatinib showed the best survival SUCRA, along with posterior HR 0.59 (0.43–0.80) and >99% posterior probability of outperforming standard chemotherapy with no maintenance (Table 1 and Fig. 3). Clinically meaningful PFS benefits were demonstrated with first‐line afatinib, gefitinib, erlotinib, and erlotinib+bevacizumab, each with maintenance regimens as illustrated in Table 1 and Appendix Figure 4.

Head‐to‐head comparisons for OS showed that first‐line afatinib with afatinib maintenance outperformed first‐line erlotinib with erlotinib maintenance as illustrated in Appendix Table 19. Head‐to‐head comparisons for PFS benefits are shown in Appendix Table 20.

##### EGFR mutation subtype L858R

In patients with EGFR mutation subtype L858R, no treatment demonstrated clinically meaningful OS benefit compared to standard chemotherapy with no maintenance (Table 1 and Fig. 3). Clinically meaningful PFS benefits were demonstrated with first‐line erlotinib, gefitinib, afatinib, and erlotinib + bevacizumab, each with maintenance regimens as illustrated in Table 1 and Appendix Figure 21.

Head‐to‐head comparisons for OS and PFS showed no strong evidence of differences among first‐line afatinib, gefitinib, erlotinib, and chemotherapy (gefitinib maintenance) as shown in Appendix Table 22.

##### EGFR mutation wild type

In EGFR wild‐type patients, first‐line chemotherapy+bevacizumab with bevacizumab maintenance was the only treatment which showed combined clinically meaningful OS and PFS benefits. First‐line chemotherapy + bevacizumab with bevacizumab maintenance showed the best survival SUCRA, along with posterior HR 0.57 (0.35–0.94**)** and 99% posterior probability of outperforming standard chemotherapy with no maintenance (Table 1 and Fig. 3). Clinically meaningful PFS benefits were demonstrated with first‐line chemotherapy + bevacizumab and chemotherapy + gefitinib, each with maintenance regimens as illustrated in Table 1 and Appendix Figure 4.

Head‐to‐head comparisons for OS showed that first‐line chemotherapy + bevacizumab with bevacizumab maintenance outperformed first‐line erlotinib + bevacizumab, gefitinib, and erlotinib, each with maintenance regimens as illustrated in Appendix Table 23. Head‐to‐head comparisons for PFS benefits are shown in Appendix Table 24.

#### EGFR FISH positive

In EGFR FISH‐positive patients with squamous histology, both first‐line chemotherapy + cetuximab with cetuximab maintenance and chemotherapy + necitumumab with necitumumab maintenance showed combined clinically meaningful OS and PFS benefits. First‐line chemotherapy + cetuximab with cetuximab maintenance and chemotherapy + necitumumab with necitumumab maintenance showed respective posterior OS HRs 0.56 (0.35–0.89) and 0.70 (0.48–1.01) with 99% and 97% posterior probabilities of outperforming standard chemotherapy with no maintenance (Appendix Table 12). On the contrary, for EGFR FISH‐positive and unselected histology, first‐line chemotherapy+cetuximab with cetuximab maintenance did not show clinically meaningful OS or PFS benefits (Appendix Table 12).

### Clinically selected populations

#### Histology

##### Nonsquamous

**Figure 4:**
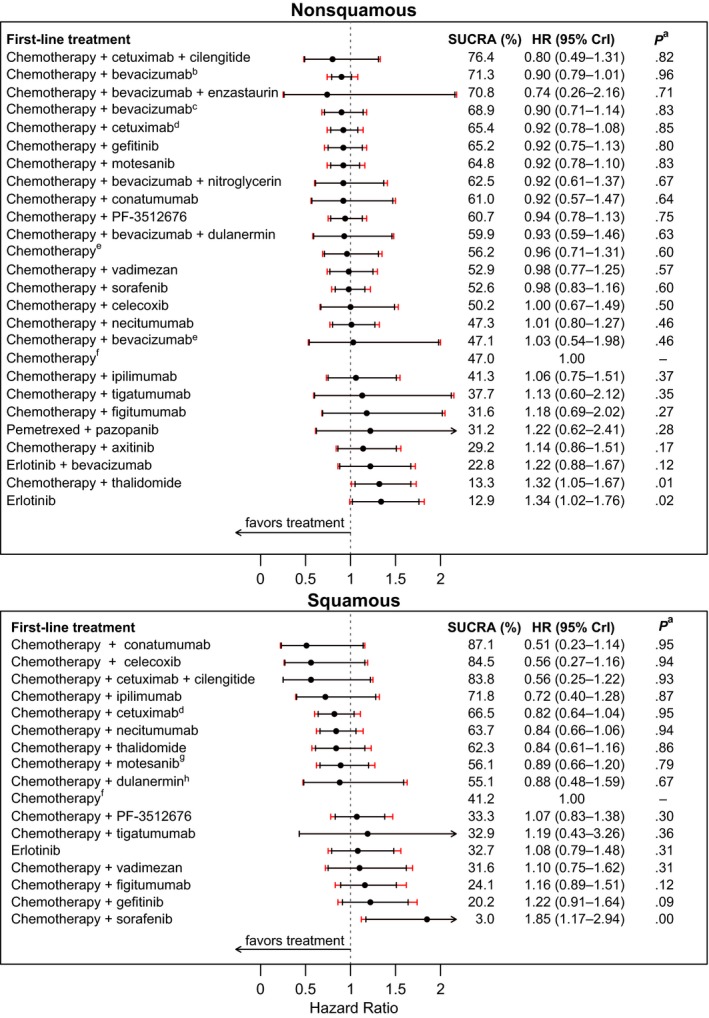
Overall survival hazard ratio (95% CrI), surface under the cumulative ranking curve (SUCRA) probability, and probability^a^ better than standard chemotherapy with no maintenance by histology (EGFR mutation unselected) for first‐line therapies with corresponding maintenance regimens. ^a^Posterior probability better than standard chemotherapy with no maintenance. ^b^Bevacizumab maintenance. ^c^Pemetrexed + bevacizumab maintenance. ^**d**^Included FLEX 38, 39, 40 with EGFR‐IHC‐positive population (≥1 cell stained positive), ^e^Pemetrexed maintenance. ^f^No maintenance. ^g^Motesanib 125 mg once daily ^h^Dulanermin 8 mg/kg.

In nonsquamous histology, no treatment demonstrated clinically meaningful OS benefit compared to standard chemotherapy with no maintenance (Table 2 and Fig. 4). Clinically meaningful PFS benefits were demonstrated with first‐line chemotherapy + bevacizumab and chemotherapy + bevacizumab+dulanermin, each with maintenance regimens as illustrated in Table 2 and Appendix Figure 5.

**Table 2:** Overall survival and progression‐free survival by histology

First‐line treatment	Maintenance treatment	Overall survival				Progression‐free survival		
		SUCRA	HR (95% CrI)	Probability better than standard chemotherapy with no maintenance	Probability best	HR (95% CrI)	Probability better than standard chemotherapy with no maintenance	Probability best
Nonsquamous								
Chemotherapy+cetuximab+cilengitide	Cetuximab+cilengitide	76.4%	0.80 (0.49–1.31)	0.82	0.19	0.76 (0.43–1.33)	0.84	0.11
Chemotherapy+bevacizumab	Bevacizumab	71.3%	0.90 (0.79–1.01)	0.96	0.00	0.74 (0.65–0.84)	1.00	0.00
Chemotherapy+bevacizumab+enzastaurin	Bevacizumab+enzastaurin	70.8%	0.74 (0.26–2.16)	0.71	0.40	0.77 (0.34–1.73)	0.74	0.18
Chemotherapy+bevacizumab	Pemetrexed+bevacizumab	68.9%	0.90 (0.71–1.14)	0.83	0.01	0.61 (0.47–0.80)	1.00	0.18
Chemotherapy+cetuximab^a^	Cetuximab	65.4%	0.92 (0.78–1.08)	0.85	0.00	0.90 (0.67–1.20)	0.77	0.00
Chemotherapy+gefitinib	Gefitinib	65.2%	0.92 (0.75–1.13)	0.80	0.01	–	–	–
Chemotherapy+motesanib	Motesanib	64.8%	0.92 (0.78–1.10)	0.83	0.00	0.81 (0.68–0.98)	0.98	0.00
Chemotherapy+bevacizumab+nitroglycerin	Bevacizumab+nitroglycerin	62.5%	0.92 (0.61–1.37)	0.67	0.05	0.94 (0.66–1.33)	0.64	0.00
Chemotherapy+conatumumab	Conatumumab	61.0%	0.92 (0.57–1.47)	0.64	0.07	1.08 (0.71–1.64)	0.36	0.00
Chemotherapy+PF‐3512676 TLR‐9 agonist	PF‐3512676 TLR‐9 agonist	60.7%	0.94 (0.78–1.13)	0.75	0.00	–	–	–
Chemotherapy+bevacizumab+dulanermin	Bevacizumab+dulanermin	59.9%	0.93 (0.59–1.46)	0.63	0.06	0.63 (0.41–0.96)	0.98	0.16
Chemotherapy	Pemetrexed	56.2%	0.96 (0.71–1.31)	0.60	0.01	0.78 (0.57–1.08)	0.94	0.01
Chemotherapy+vadimezan	Vadimezan	52.9%	0.98 (0.77–1.25)	0.57	0.00	–	–	–
Chemotherapy+sorafenib	Sorafenib	52.6%	0.98 (0.83–1.16)	0.60	0.00	0.87 (0.73–1.03)	0.96	0.00
Chemotherapy+celecoxib	Celecoxib	50.2%	1.00 (0.67–1.49)	0.50	0.02	0.91 (0.62–1.33)	0.69	0.01
Chemotherapy+necitumumab	Necitumumab	47.3%	1.01 (0.80–1.27)	0.46	0.00	0.96 (0.75–1.24)	0.63	0.00
Chemotherapy+bevacizumab	Pemetrexed	47.1%	1.03 (0.54–1.98)	0.46	0.07	0.84 (0.46–1.53)	0.72	0.05
Chemotherapy	No maintenance	47.0%	1.00	–	0.00	1.00	–	0.00
Chemotherapy+ipilimumab	Ipilimumab	41.3%	1.06 (0.75–1.51)	0.37	0.01	0.84 (0.60–1.19)	0.84	0.01
Chemotherapy+tigatuzumab	Tigatuzumab	37.7%	1.13 (0.60–2.12)	0.35	0.04	0.84 (0.46–1.53)	0.72	0.07
Chemotherapy+figitumumab	Figitumumab	31.6%	1.18 (0.69–2.02)	0.27	0.02	–	–	–
Pemetrexed+pazopanib	Pazopanib	31.2%	1.22 (0.62–2.41)	0.28	0.03	0.75 (0.42–1.34)	0.84	0.13
Chemotherapy+axitinib	Axitinib	29.2%	1.14 (0.86–1.51)	0.17	0.00	0.90 (0.67–1.21)	0.76	0.00
Erlotinib+bevacizumab	Erlotinib+bevacizumab	22.8%	1.22 (0.88–1.67)	0.12	0.00	1.40 (1.01–1.93)	0.02	0.00
Chemotherapy+thalidomide	Thalidomide	13.3%	1.32 (1.05–1.67)	0.01	0.00	1.26 (0.98–1.62)	0.03	0.00
Erlotinib	Erlotinib	12.9%	1.34 (1.02–1.76)	0.02	0.00	1.50 (1.15–1.96)	0.00	0.00
Chemotherapy+cediranib	Cediranib	–	–	–	–	0.89 (0.56–1.41)	0.69	0.02
Squamous								
Chemotherapy+conatumumab	Conatumumab	87.1%	0.51 (0.23–1.14)	0.95	0.36	0.47 (0.22–1.00)	0.98	0.23
Chemotherapy+celecoxib	Celecoxib	84.5%	0.56 (0.27–1.16)	0.94	0.26	0.56 (0.28–1.10)	0.96	0.10
Chemotherapy+cetuximab+cilengitide	Cetuximab+cilengitide	83.8%	0.56 (0.25–1.22)	0.93	0.27	0.36 (0.13–1.00)	0.98	0.53
Chemotherapy+ipilimumab	Ipilimumab	71.8%	0.72 (0.40–1.28)	0.87	0.06	0.61 (0.34–1.10)	0.95	0.05
Chemotherapy+cetuximab^a^	Cetuximab	66.5%	0.82 (0.64–1.04)	0.95	0.00	0.70 (0.44–1.10)	0.94	0.00
Chemotherapy+necitumumab	Necitumumab	63.7%	0.84 (0.66–1.06)	0.94	0.00	0.85 (0.66–1.10)	0.91	0.00
Chemotherapy+thalidomide	Thalidomide	62.3%	0.84 (0.61–1.16)	0.86	0.00	0.84 (0.60–1.18)	0.85	0.00
Chemotherapy+motesanib	Motesanib	56.1%	0.89 (0.66–1.20)	0.79	0.00	0.85 (0.61–1.19)	0.84	0.00
Chemotherapy+dulanermin^b^	Dulanermin^b^	55.1%	0.88 (0.48–1.59)	0.67	0.02	1.12 (0.65–1.94)	0.33	0.00
Chemotherapy	No maintenance	41.2%	1.00	–	0.00	1.00	–	0.00
Chemotherapy+PF‐3512676 TLR‐9 agonist	PF‐3512676 TLR‐9 agonist	33.3%	1.07 (0.83–1.38)	0.30	0.00	–	–	–
Chemotherapy+tigatumumab	Tigatumumab	32.9%	1.19 (0.43–3.26)	0.36	0.02	0.90 (0.33–2.45)	0.58	0.03
Erlotinib	Erlotinib	32.7%	1.08 (0.79–1.48)	0.31	0.00	1.56 (1.15–2.12)	0.00	0.00
Chemotherapy+vadimezan	Vadimezan	31.6%	1.10 (0.75–1.62)	0.31	0.00	–	–	–
Chemotherapy+figitumumab	Figitumumab	24.1%	1.16 (0.89–1.51)	0.12	0.00	–	–	–
Chemotherapy+gefitinib	Gefitinib	20.2%	1.22 (0.91–1.64)	0.09	0.00	–	–	–
Chemotherapy+sorafenib	Sorafenib	3.0%	1.85 (1.17–2.94)	0.00	0.00	1.31 (0.88–1.94)	0.09	0.00
Chemotherapy+cediranib	Cediranib	–	–	–	–	0.66 (0.32–1.35)	0.88	0.05

HR, hazard ratio; CrI, credible intervals; TLR, toll‐like receptor.

a Included FLEX [38, 39, 40] with EGFR‐IHC‐positive population (≥ 1 cell stained positive).

b Dulanermin 8 mg/kg.

##### Squamous

In squamous histology, first‐line chemotherapy+conatumumab with conatumumab maintenance was the only treatment which showed combined clinically meaningful OS and PFS benefits. First‐line chemotherapy+conatumumab with conatumumab maintenance showed best survival SUCRA, along with posterior HR 0.51 (0.23–1.14) and 95% posterior probability of outperforming standard chemotherapy with no maintenance (Table 2 and Fig. 4). Clinically meaningful PFS benefits were demonstrated with first‐line chemotherapy + conatumumab, chemotherapy + celecoxib, chemotherapy + cetuximab + cilengitide, and chemotherapy + ipilimumab, each with maintenance regimens as illustrated in Table 2 and Appendix Figure 5.

### Clinically enriched for EGFR mutations

In patients clinically enriched for EGFR mutations with nonsquamous histology, both first‐line chemotherapy+bevacizumab with bevacizumab maintenance and sequential chemotherapy + gefitinib with gefitinib maintenance showed combined clinically meaningful OS and PFS benefits. First‐line chemotherapy + bevacizumab with bevacizumab maintenance and sequential chemotherapy + gefitinib with gefitinib maintenance showed respective posterior HRs 0.78 (0.59–1.05) and 0.79 (0.60–1.05), both with 95% posterior probabilities of outperforming standard chemotherapy with no maintenance (Appendix Table 13). Clinically meaningful PFS benefits were demonstrated with first‐line chemotherapy + bevacizumab with bevacizumab maintenance, sequential chemotherapy + gefitinib with gefitinib maintenance, and intercalated chemotherapy + erlotinib with erlotinib maintenance, each with maintenance regimens as illustrated in Appendix Table 13.

## Discussion

This meta‐analysis showed combined clinically meaningful OS and PFS benefits of particular first‐line with maintenance treatments in advanced NSCLC patients *selected by molecular and/or clinical biomarkers*. Results suggest the following treatment and patient selection strategies; (a) for molecularly selected patients, the following showed combined clinically meaningful OS and PFS benefits; (i) first‐line intercalated chemotherapy+erlotinib, maintenance erlotinib in patients with EGFR mutations, (ii) first‐line afatinib, maintenance afatinib in patients with EGFR deletion 19, (iii) first‐line chemotherapy+bevacizumab, maintenance bevacizumab in EGFR wild‐type patients, and (iv) first‐line chemotherapy+cetuximab, maintenance cetuximab or first‐line chemotherapy+necitumumab, maintenance necitumumab in EGFR FISH‐positive patients with squamous histology, whereas (b) for clinically selected patients, the following showed combined clinically meaningful OS and PFS benefits; (i) first‐line chemotherapy+conatumumab, maintenance conatumumab in patients with squamous histology and (ii) first‐line chemotherapy + bevacizumab, maintenance bevacizumab or first‐line sequential chemotherapy + gefitinib, maintenance gefitinib in patients clinically enriched for EGFR mutations with nonsquamous histology. No treatment showed combined clinically meaningful OS and PFS benefits in patients with EGFR L858R or nonsquamous histology.

This meta‐analysis highlights the importance of testing for specific subtypes of EGFR mutation (Del19/L858R) as results suggest that deletion 19 and L858R could be clinically distinct and exhibit different treatment outcomes. Feasibility of wide‐spread EGFR testing has been greatly extended by recent advances in ‘liquid biopsy’ or plasma‐based genotyping 26, 27. In particular, plasma‐based genotyping has been shown to detect both EGFR deletion 19 and L858R rapidly and accurately, reducing the need for traditional invasive biopsies 26, 27. Further trials in the EGFR mutation setting should study treatments distinctly by subtype. Urgent research is needed to identify treatments that will benefit patients with L858R subtype as they make up approximately 40% of identified EGFR mutations 14. This study has found little evidence of effective treatments for patients with this subtype.

When interpreting OS benefit of first‐line intercalated chemotherapy+erlotinib in patients with EGFR mutations, it is worth noting that result was derived from exploratory analysis of a single trial (FASTACT‐2) 28. Additionally, biomarker analyses revealed that the majority of this study population had 23% EGFR mutation Del 19 and 14% L858R, which might corroborate evidence from this meta‐analysis on the preferential OS benefit in Del 19 compared to L858R, although confirmatory studies are needed 29. Furthermore, only 57% of the trial population was tested and testing was not mandatory. Hence, results may not be representative of the full trial population 29.

Previous studies in the maintenance setting have suggested survival benefits with maintenance pemetrexed in nonsquamous patients who did not progress after first‐line cisplatin/pemetrexed 4, 30. However, in this meta‐analysis of first‐line trials followed by maintenance regimens, no clinically meaningful survival benefit was observed for patients with nonsquamous histology. It is important to note that in the earlier maintenance trial 30, only patients who had disease control were randomized to a maintenance therapy. In this study all patients randomized to first‐line therapy contributed to comparative estimates of first‐line with maintenance regimens, whereas only patients with disease control were given maintenance therapy. The PRONOUNCE trial, for example, contained 42% disease progressors 31. This raises an important question for future research, namely, is first‐line cisplatin/pemetrexed with pemetrexed maintenance beneficial for all nonsquamous patients, or are its benefits limited to patients with disease control after first‐line therapy.

In this study, aggregated data meta‐analysis, as opposed to individual patient data meta‐analysis, was performed. However, we believe that findings are robust due in particular to the systematic selection of well‐designed trials and objective outcomes. An additional limitation of this study is that trials which had no common comparator arms could not be included within the network of comparisons, for example, oral versus intravenous vinorelbine as single first‐line agent 32 or first‐line chemotherapy with maintenance vinorelbine versus gemcitabine 33.

Notably, the level of uncertainty (and corresponding power to detect differences, if present) varied across comparisons, and was driven by several factors, including the number of studies informing the comparison, the level of uncertainty/sample sizes of the relevant studies, the estimate of study‐to‐study heterogeneity in the efficacy of treatments, and how indirect the evidence pertaining to the specific comparison was. In particular, some comparisons were relatively uncertain, as reflected by wide credible and predictive intervals, and this uncertainty was a function of the data used for analysis. The analysis data were constrained in two ways, by availability and by inclusion criteria. Data availability reflected both population sizes of disease subgroups and current clinical thinking. Here, the inclusion criteria balance inclusiveness with transparency and robustness of modeling and results.

In particular, several treatments within subgroups failed to show a combined clinically meaningful benefit, but this does not necessary mean that there is evidence that the treatment is not beneficial. Importantly, if the comparison of interest was uncertain, then there may not have been enough evidence to make strong conclusions of any kind, such as concluding that there was a combined clinically meaningful benefit. Consider, for example, results for the nonsquamous subgroup. Several treatments showed some degree of promise, but none crossed the combined clinically meaningful benefit thresholds, which measure both the size of effect (hazard ratio) and uncertainty (probability better than standard chemotherapy with no maintenance). More broadly, several of the treatments within subgroups which showed promise, but failed to achieve combined clinically meaningful benefit, in particular those for which results were quite uncertain, may warrant further investigation.

To our knowledge, this study is the first multiple treatment comparison meta‐analysis to evaluate efficacies of first‐line therapies with maintenance regimens head‐to‐head and elucidate treatments with combined clinically meaningful OS and PFS benefits in patients selected by molecular and clinical biomarkers. An earlier meta‐analysis has shown clinically meaningful survival benefits of maintenance treatments in advanced NSCLC 4. However, those maintenance trials randomized only nonprogressing patients after first‐line chemotherapy rendering pooling of evidence with studies in the current meta‐analysis inappropriate due to differences in the distribution of disease trajectories of patients between the two distinct study designs.

Recently, first‐line immunotherapies as a monotherapy have been tested in PD‐L1‐positive advanced NSCLC 34, 35. Interestingly, pembrolizumab showed improved OS in patients with high PD‐L1 expression. (≥50%), whereas nivolumab showed no survival benefits in patients with low PD‐L1 expression (≥1%) 34, 35. Further research on strategies to identify patients who will have the greatest benefit from immunotherapies in the first‐line setting of advanced NSCLC is needed. In addition, a trial is currently underway to compare efficacy of the third‐generation EGFR TKI osimertinib with first‐generation erlotinib/gefitinib as monotherapy in the first‐line setting of activating EGFR mutant advanced NSCLC 36. When data from these promising studies become available in the future, it will add to the knowledge and evidence base of treatment options and patient selection strategies for improving treatment outcomes in advanced NSCLC patients.

In conclusion, this meta‐analysis of current evidence shows that particular first‐line with maintenance treatments show clinically meaningful OS and PFS benefits in molecularly and/or clinically selected populations. Further research is needed to achieve effective therapy for patients with EGFR mutation L858R or nonsquamous histology.

## Conflict of Interest

GL reports grants and personal fees from Roche, grants and personal fees from Sanofi Aventis, grants and personal fees from Astra Zeneca, grants and personal fees from Boehringer Ingelheim, grants and personal fees from Lilly, grants and personal fees from Merck Serono, grants and personal fees from Merck, Sharp and Dhome, and grants and personal fees from BMS, outside the submitted work.

## Supporting information


**Appendix S1.** Supplementary Tables and Figures.
