# Supplementary material for: Meta‐analysis of first‐line therapies with maintenance regimens for advanced non‐small‐cell lung cancer (NSCLC) in molecularly and clinically selected populations
Source: Cancer Med. 2017 Jul 3;6(8):1847–60. doi: 10.1002/cam4.1101 (PMC5548880; doi:10.1002/cam4.1101)
Supplement: Supplementary file 1 — Appendix S1. Supplementary Tables and Figures. [file CAM4-6-1847-s001.pdf]

Appendix

Appendix Table 1: Characteristics of studies evaluating first-line therapies followed by maintenance regimens in advanced NSCLC patients

	Study	Population	First-line treatment	Maintenance treatment[a]	N	Median OS (months)
1	BEYOND[1,2]	Treatment naïve nonsquamous stage IIIb/IV or recurrent NSCLC with ECOG PS 0-1 in the Chinese population	Carboplatin AUC 6 + paclitaxel 175 mg/m^2 + bevacizumab 15 mg/kg day 1 every 21 days (6 cycles)	Bevacizumab 15 mg/kg every 21 days	138	24.3
			Carboplatin AUC 6 + paclitaxel 175 mg/m^2 + placebo day 1 every 21 days (6 cycles)	Placebo every 21 days	138	17.7
2	INNOVATIONS[3]	Treatment naïve nonsquamous stage IIIb/IV NSCLC with ECOG PS 0-2	Erlotinib 150 mg daily + bevacizumab 15 mg/kg day 1 every 21 days	Erlotinib 150 mg daily + bevacizumab 15 mg/kg day 1 every 21 days	111	12.6
			Cisplatin 80 mg/m^2 day 1 + gemcitabine 1250 mg/m^2 days 1 and 8 + bevacizumab 15 mg/kg day 1 every 21 days (6 cycles)	Bevacizumab 15 mg/kg day 1 every 21 days	113	17.7
3	EURTAC[4–7]	Chemonaïve stage IIIb/IV NSCLC with ECOG PS 0-2 harboring activating EGFR mutations (exon 19 deletion or exon 21 L858R) in European population	Erlotinib 150 mg daily	Erlotinib 150 mg daily	86	22.9
			Cisplatin 75 mg/m^2 day 1 + docetaxel 75 mg/m^2 day 1 or cisplatin 75 mg/m^2 day 1 + gemcitabine 1250 mg/m^2 days 1 and 8 or carboplatin AUC 6 day 1	No maintenance[c]	88	19.6

			+ docetaxel 75 mg/m^2 day 1 or carboplatin AUC 5 day 1 + gemcitabine 1000 mg/m^2 days 1 and 8 every 21 days (4 cycles)			
4	TORCH[8]	Treatment naïve stage IIIb/IV NSCLC with ECOG PS 0-1 (unselected by EGFR mutation) in predominantly Caucasian population	Erlotinib 150 mg daily	Erlotinib 150 mg daily[d]	380	8.7
			Cisplatin 80 mg/m^2 day 1 + gemcitabine 1,200 mg/m^2 days 1 and 8 every 21 days (6 cycles)	No maintenance[d]	380	11.6
5	OPTIMAL[6,9–12]	Treatment naïve stage IIIb/IV or recurrent NSCLC with ECOG PS 0-2 harboring activating EGFR mutations (exon 19 deletion or exon 21 L858R) in the Chinese population	Erlotinib 150 mg daily	Erlotinib 150 mg daily	82	22.8
			Carboplatin AUC 5 day 1 + gemcitabine 1000 mg/m^2 days 1 and 8 every 21 days (4 cycles)	No maintenance	72	27.2
6	ENSURE[6,13–15]	Treatment naïve stage IIIb/IV NSCLC with ECOG PS 0-2 harboring EGFR mutations in the Asian population	Erlotinib 150 mg daily	Erlotinib 150 mg daily	110	26.3
			Cisplatin 75 mg/m^2 day 1 + gemcitabine 1,250 mg/m^2 days 1 and 8 every 21 days (4 cycles)	No maintenance	107	25.5
7	IPASS[16–18]	Treatment naïve stage IIIb/IV NSCLC adenocarcinoma	Gefitinib 250 mg daily	Gefitinib 250 mg daily	609	18.8

		with WHO PS 0-2 who were non or previous light smokers in the East-Asian population				
			Carboplatin AUC 5 or 6 + paclitaxel 200 mg/m^2 every 21 days (6 cycles)	No maintenance	608	17.4
8	NEJ002[6,19,20]	Chemonaïve stage IIIb/IV or recurrent NSCLC with ECOG PS 0-1 harboring activating EGFR mutations in the Japanese population[e]	Gefitinib 250 mg daily	Gefitinib 250 mg daily	114	27.7
			Carboplatin AUC 6 + paclitaxel 200 mg/m^2 day 1 every 21 days (3-6 cycles)	No maintenance	114	26.6
9	WJTOG3405[6,21,22]	Chemonaïve stage IIIb/IV or recurrent NSCLC with WHO PS 0-1 harboring activating EGFR mutations (exon 19 deletion or exon 21 L858R) in the Japanese population	Gefitinib 250 mg daily	Gefitinib 250 mg daily	86	34.8
			Cisplatin 80 mg/m^2 + docetaxel 60 mg/m^2 every 21 days (3-6 cycles)	No maintenance	86	37.3
10	First-SIGNAL[23]	Chemonaïve stage IIIb/IV adenocarcinoma NSCLC with ECOG PS 0-2 who were never smokers in the Korean population	Gefitinib 250 mg daily	Gefitinib 250 mg daily	159	22.3
			Cisplatin 80 mg/m^2 day 1 + gemcitabine 1,250 mg/m^2 days 1 and 8 every 21 days (9 cycles)	No maintenance	150	22.9
11	LUX-Lung-3 [6,24–26]	Treatment naïve stage IIIb/IV lung	Afatinib 40 mg daily	Afatinib 40 mg daily	230	28.2

		adenocarcinoma with ECOG PS 0-1 harboring activating EGFR mutations[f]				
			Cisplatin 75 mg/m^2 + pemetrexed 500 mg/m^2 every 21 days (6 cycles)	No maintenance	115	28.2
12	LUX-Lung-6 [6,25–27]	Treatment naïve stage IIIb/IV NSCLC adenocarcinoma with ECOG PS 0-1 harboring EGFR mutations in the Asian population	Afatinib 40 mg daily	Afatinib 40 mg daily	242	23.1
			Cisplatin 75 mg/m^2 day 1 + gemcitabine 1000 mg/m^2 days 1 and 8 every 21 days (6 cycles)	No maintenance	122	23.5
13	LUX-Lung-7[28]	Treatment naïve stage IIIb/IV NSCLC adenocarcinoma with ECOG PS 0-1 harboring EGFR mutations (exon 19 deletion and/or L858R)	Afatinib 40 mg daily	Afatinib 40 mg daily	160	-
			Gefitinib 250 mg daily	Gefitinib 250 mg daily	159	-
14	TRIBUTE[29,30]	Treatment naïve stage IIIb/IV NSCLC with ECOG PS 0-1[g]	Carboplatin AUC 6 + paclitaxel 200 mg/m^2 every 21 days (6 cycles) + erlotinib 150 mg daily	Erlotinib 150 mg daily	539	10.6
			Carboplatin AUC 6 + paclitaxel 200 mg/m^2 every 21 days (6 cycles) + placebo daily	Placebo daily	540	10.5
15	INTACT 1[31,32]	Chemonaïve unresectable stage III/IV NSCLC with WHO PS 0-2[b]	Cisplatin 80 mg/m^2 day 1 + gemcitabine 1,250 mg/m^2 days 1 and 8 every 21 days (6 cycles) +	Gefitinib 500 mg daily	365	9.9

			gefitinib 500 mg daily			
			Cisplatin 80 mg/m^2 day 1 + gemcitabine 1,250 mg/m^2 days 1 and 8 every 21 days (6 cycles) + gefitinib 250 mg daily	Gefitinib 250 mg daily	365	9.9
			Cisplatin 80 mg/m^2 day 1 + gemcitabine 1,250 mg/m^2 days 1 and 8 every 21 days (6 cycles) + placebo	Placebo daily	363	10.9
16	INTACT 2 [b 32,33]	Chemonaïve unresectable stage III/IV NSCLC with WHO PS 0-2	Carboplatin AUC 6 + paclitaxel 225 mg/m^2 day 1 every 21 days (6 cycles) + gefitinib 500 mg daily	Gefitinib 500 mg daily	347	8.7
			Carboplatin AUC 6 + paclitaxel 225 mg/m^2 day 1 every 21 days (6 cycles) + gefitinib 250 mg daily	Gefitinib 250 mg daily	345	9.8
			Carboplatin AUC 6 + paclitaxel 225 mg/m^2 day 1 every 21 days (6 cycles) + placebo daily	Placebo daily	345	9.9
17	FASTACT-2[34]	Treatment naïve stage IIIb/IV NSCLC with ECOG PS 0-1 in the Asian population	Platinum (carboplatin AUC 5 or cisplatin 75 mg/m^2) day 1 + gemcitabine 1,250 mg/m^2 days 1 and 8 + erlotinib 150 mg daily days 15-28 every 28 days (6 cycles)	Erlotinib 150 mg daily days 15-28 every 28 days	226	18.3
			Platinum (carboplatin AUC 5 or cisplatin 75 mg/m^2) day 1	Placebo daily days 15-28 every 28 days	225	15.2

			+ gemcitabine 1,250 mg/m^2 days 1 and 8 + placebo days 15-28 every 28 days (6 cycles)			
18	Yang et al.[35,36]	Chemonaïve advanced nonsquamous NSCLC with ECOG PS 0-1 who were non or previous light smokers in the East Asian population	Cisplatin 75 mg/m^2 + pemetrexed 500 mg/m^2 day 1 every 21 days (6 cycles)	Gefitinib 250 mg daily	118	26.87
			Gefitinib 250 mg daily	Gefitinib 250 mg daily	118	27.86
19	JO25567[37,38]	Chemonaïve stage IIIb/IV or recurrent nonsquamous NSCLC with ECOG PS 0-1 harboring activating EGFR mutations (exon 19 deletion or exon 21 L858R) in the Japanese population	Erlotinib 150 mg daily + bevacizumab 15 mg/kg day 1 every 21 days	Erlotinib 150 mg daily + bevacizumab 15 mg/kg day 1 every 21 days	75	-
			Erlotinib 150 mg daily	Erlotinib 150 mg daily	77	-
20	FLEX[39–41]	Chemonaïve stage IIIb/IV NSCLC with ECOG PS 0-2 and EGFR-IHC positive ($\geq$ 1 cell stained positive)	Cisplatin 80 mg/m^2 day 1 + vinorelbine 25 mg/m^2 days 1 and 8 every 21 days (6 cycles) + cetuximab 400 mg/m^2 initial dose day 1 followed by 250 mg/m^2 weekly onwards	Cetuximab 250 mg/m^2 weekly	557	11.3
			Cisplatin 80 mg/m^2 day 1 + vinorelbine 25 mg/m^2 days 1 and 8 every 21 days (6 cycles)	No maintenance	568	10.1
21	Cheng et al.[42]	Chemonaïve stage IV nonsquamous NSCLC with ECOG PS 0-1 harboring activating EGFR	Gefitinib 250 mg daily + pemetrexed 500 mg/m^2 every 21 days	Gefitinib 250 mg daily + pemetrexed 500 mg/m^2 every 21 days	126	-

		mutations in the East-Asian population				
			Gefitinib 250 mg daily	Gefitinib 250 mg daily	65	
22	TASK[43]	Treatment naïve nonsquamous recurrent or stage IIIb/IV NSCLC with ECOG PS 0-1	Erlotinib 150 mg daily + bevacizumab 15 mg/kg day 1 every 21 days	Erlotinib 150 mg daily + bevacizumab 15 mg/kg day 1 every 21 days	63	16.4
			Chemotherapy doublet every 21 days (cisplatin 80 mg/m^2 day 1 + gemcitabine 1,250 mg/m^2 days 1 and 8 or carboplatin AUC 6 + paclitaxel 200 mg/m^2 day 1) + bevacizumab 15 mg/kg day 1 every 21 days (4-6 cycles)	Bevacizumab 15 mg/kg day 1 every 21 days	61	Not reached
23	ECOG 4599[44]	Chemonaïve nonsquamous stage IIIb/IV or recurrent NSCLC with ECOG PS 0-1 in predominantly Caucasian population	Carboplatin AUC 6 + paclitaxel 200 mg/m^2 + bevacizumab 15 mg/kg day 1 every 21 days (6 cycles)	Bevacizumab 15 mg/kg every 21 days	417	12.3
			Carboplatin AUC 6 + paclitaxel 200 mg/m^2 day 1 every 21 days (6 cycles)	No maintenance	433	10.3
24	JO19907[45]	Chemonaïve nonsquamous stage IIIb/IV or recurrent NSCLC with ECOG PS 0-1 in the Japanese population	Carboplatin AUC 6 + paclitaxel 200 mg/m^2 + bevacizumab 15 mg/kg day 1 every 21 days (6 cycles)	Bevacizumab 15 mg/kg every 21 days	117	22.8

			Carboplatin AUC 6 + paclitaxel 200 mg/m^2 day 1 every 21 days (6 cycles)	No maintenance	58	23.4
25	PRONOUNCE[46,47]	Chemonaïve nonsquamous stage IV NSCLC with ECOG PS 0-1	Carboplatin AUC 6 + pemetrexed 500 mg/m^2 every 21 days (4 cycles)	Pemetrexed 500 mg/m^2 every 21 days	182	10.5
			Carboplatin AUC 6 + paclitaxel 200 mg/m^2 + bevacizumab 15 mg/kg every 21 days (4 cycles)	Bevacizumab 15 mg/kg every 21 days	179	11.7
26	PointBreak[48,49]	Treatment naïve nonsquamous stage IIIb/IV NSCLC with ECOG PS 0-1	Carboplatin AUC 6 + pemetrexed 500 mg/m^2 + bevacizumab 15 mg/kg day 1 every 21 days (4 cycles)	Pemetrexed 500 mg/m^2 + bevacizumab 15 mg/kg every 21 days	472	12.6
			Carboplatin AUC 6 + paclitaxel 200 mg/m^2 + bevacizumab 15 mg/kg day 1 every 21 days (4 cycles)	Bevacizumab 15 mg/kg every 21 days	467	13.4
27	INSPIRE[50]	Treatment naïve stage IV nonsquamous NSCLC with ECOG PS 0-2	Cisplatin 75 mg/m^2 + pemetrexed 500 mg/m^2 day 1 + necitumumab 800 mg days 1 and 8 every 21 days (6 cycles)	Necitumumab 800 mg days 1 and 8 every 21 days	315	11.3
			Cisplatin 75 mg/m^2 + pemetrexed 500 mg/m^2 day 1 every 21 days (6 cycles)	No maintenance	318	11.5
28	Paz-Ares et al.[51]	Treatment naïve stage IIIb/IV or recurrent NSCLC with ECOG PS 0-1	Carboplatin AUC 6 + paclitaxel 200 mg/m^2 + conatumumab	Conatumumab 3 mg/kg day 1 every 21 days	57	12.3

			3 mg/kg day 1 every 21 days (6 cycles)			
			Carboplatin AUC 6 + paclitaxel 200 mg/m^2 + conatumumab 15 mg/kg day 1 every 21 days (6 cycles)	Conatumumab 15 mg/kg day 1 every 21 days	56	11.4
			Carboplatin AUC 6 + paclitaxel 200 mg/m^2 + placebo day 1 every 21 days (6 cycles)	Placebo day 1 every 21 days	59	7.8
29	Reck et al.[52]	Treatment naïve stage IIIb/IV NSCLC with ECOG PS 0-1 (included 1/97 stage IIb)	Carboplatin AUC 6 + paclitaxel 175 mg/m^2 every 21 days + tigatuzumab 10 mg/kg week 1 of first cycle and 8 mg/kg following cycles once every 21 days (6 cycles)	Tigatuzumab 8 mg/kg every 21 days	49	8.4
			Carboplatin AUC 6 + paclitaxel 175 mg/m^2 + placebo every 21 days (6 cycles)	Placebo every 21 days	48	9.0
30	NVALT-4[53]	Chemonaïve stage IIIb/IV NSCLC with WHO PS 0-2	Carboplatin AUC 6 + docetaxel 75 mg/m^2 day 1 every 21 days (5 cycles) + celecoxib 400 mg twice daily	Celecoxib 400 mg twice daily (maximum 3 years)	281	8.2
			Carboplatin AUC 6 + docetaxel 75 mg/m^2 day 1 every 21 days (5 cycles) + placebo twice daily	No maintenance	280	8.2
31	LUN06-116[54]	Treatment naïve nonsquamous stage	Carboplatin AUC 6 + pemetrexed	Bevacizumab 15 mg/kg every 21 days	20	9.1

		IIIb/IV NSCLC with ECOG PS 0-1	500 mg/m^2 + bevacizumab 15 mg/kg day 7 every 21 days (4 cycles) + enzastaurin 500 mg daily (enzastaurin 375 mg three times as loading dose on day 1)	+ enzastaurin 500 mg daily		
			Carboplatin AUC 6 + pemetrexed 500 mg/m^2 + bevacizumab 15 mg/kg day 7 every 21 days (4 cycles) + placebo daily (placebo three times as loading dose on day 1)	Bevacizumab 15 mg/kg every 21 days + placebo daily	20	7.6
32	NVALT 12[55–58]	Chemonaïve nonsquamous stage IV NSCLC with ECOG WHO PS 0-2 (allowed prior treatment with EGFR TKI)	Carboplatin AUC 6 + paclitaxel 200 mg/m^2 + bevacizumab 15 mg/kg day 1 + nitroglycerin patch 15 mg per 24 hours (day -2 to +2) every 21 days (4 cycles)	Bevacizumab 15 mg/kg day 1 + nitroglycerin patch 15 mg per 24 hours (day -2 to +2) every 21 days	111	9.4
			Carboplatin AUC 6 + paclitaxel 200 mg/m^2 + bevacizumab 15 mg/kg day 1 every 21 days (4 cycles)	Bevacizumab 15 mg/kg day 1 every 21 days	112	11.6
33	Lee et al.[59,60]	Treatment naïve stage IIIb/IV NSCLC with ECOG PS 0-1 tested negative for pregnancy	Carboplatin AUC 5 or 6 day 1 + gemcitabine 1,200 mg/m^2 days 1 and 8 every 21 days (4 cycles) + thalidomide daily (initial dose 100mg	Thalidomide daily for 2 years from start of chemotherapy	372	8.5

			daily, if tolerated, increased to 150 mg daily for a month at end of chemotherapy, and then to 200 mg daily thereafter)			
			Carboplatin AUC 5 or 6 day 1 + gemcitabine 1,200 mg/m^2 days 1 and 8 every 21 days (4 cycles) + placebo daily	Placebo daily for 2 years from start of chemotherapy	350	8.9
34	Langer et al.[61]	Treatment naïve stage IIIb/IV or recurrent nonadenocarcinoma NSCLC with ECOG PS 0-1	Carboplatin AUC 6 + paclitaxel 200 mg/m^2 day 1 every 21 days (6 cycles) + figitumumab 20 mg/kg day 1 every 21 days	Figitumumab 20 mg/kg day 1 every 21 days (17 cycles from start of chemotherapy or until progression or intolerance)	342	8.6
			Carboplatin AUC 6 + paclitaxel 200 mg/m^2 day 1 every 21 days (6 cycles)	No maintenance	339	9.8
35	ATTRACT-1[62]	Treatment naïve stage IIIb/IV NSCLC with WHO PS 0-1	Carboplatin AUC 6 + paclitaxel 200 mg/m^2 + ASA404 1,800 mg/m^2 day 1 every 21 days (6 cycles)	ASA404 1,800 mg/m^2 day 1 every 21 days	649	13.4
			Carboplatin AUC 6 + paclitaxel 200 mg/m^2 + placebo day 1 every 21 days (6 cycles)	Placebo day 1 every 21 days	650	12.7
36	Scagliotti et al.[63]	Treatment naïve nonsquamous stage IIIb/IV NSCLC with ECOG PS 0-1 (1/97 PS2)	Pazopanib 800 mg once daily + pemetrexed 500 mg/m^2 every 21 days (6 cycles)	Pazopanib once daily	62	-
			Cisplatin 75 mg/m^2 +	No maintenance	35	-

			pemetrexed 500 mg/m^2 every 21 days (6 cycles)			
37	Twelves et al.[64]	Treatment naïve nonsquamous stage IIIb/IV or recurrent NSCLC with ECOG PS 0-1	Carboplatin AUC 6 + paclitaxel 200 mg/m^2 day 1 every 21 days + axitinib 5 mg twice daily (6 cycles)	Axitinib 5 mg twice daily	58	10.6
			Carboplatin AUC 6 + paclitaxel 200 mg/m^2 + bevacizumab 15 mg/kg day 1 every 21 days (6 cycles)	Bevacizumab 15 mg/kg day 1 every 21 days	60	13.3
38	ESCAPE[65]	Chemonaïve stage IIIb/IV NSCLC with ECOG PS 0-1	Carboplatin AUC 6 + paclitaxel 200 mg/m^2 day 1 + sorafenib 400 mg twice daily days 2-19 every 21 days (6 cycles)	Sorafenib 400 mg twice daily	464	10.7
			Carboplatin AUC 6 + paclitaxel 200 mg/m^2 day 1 + placebo twice daily days 2-19 every 21 days (6 cycles)	Placebo twice daily	462	10.6
39	NExUS[66]	Chemonaïve nonsquamous stage IIIb/IV NSCLC with ECOG PS 0-1	Cisplatin 75 mg/m^2 day 1 + gemcitabine 1,250 mg/m^2 days 1 and 8 every 21 days (6 cycles) + sorafenib 400 mg twice daily	Sorafenib 400 mg twice daily	385	12.4
			Cisplatin 75 mg/m^2 day 1 + gemcitabine 1,250 mg/m^2 days 1 and 8 every 21 days (6 cycles) + placebo twice daily	Placebo twice daily	387	12.5

40	MONET1[67]	Treatment naïve nonsquamous stage IIIb/IV or recurrent NSCLC with ECOG PS 0-1	Carboplatin AUC 6 + paclitaxel 200 mg/m^2 day 1 every 21 days (6 cycles) + motesanib 125 mg once daily	Motesanib 125 mg daily	541	13.0
			Carboplatin AUC 6 + paclitaxel 200 mg/m^2 day 1 every 21 days (6 cycles) + placebo daily	Placebo daily	549	11.0
41	MONET1[68] (Unacceptable early toxicity reported)	Treatment naïve squamous stage IIIb/IV or recurrent NSCLC with ECOG PS 0-1	Carboplatin AUC 6 + paclitaxel 200 mg/m^2 day 1 every 21 days (6 cycles) + motesanib 125 mg once daily	Motesanib 125 mg daily (maximum 36 months)	182	11.1
			Carboplatin AUC 6 + paclitaxel 200 mg/m^2 day 1 every 21 days (6 cycles) + placebo daily	Placebo daily	178	10.7
42	NCIC CTG BR.24[69,70]	Treatment naïve stage IIIb/IV NSCLC with ECOG PS 0-1	Carboplatin AUC 6 + paclitaxel 200 mg/m^2 every 21 days (6-8 cycles) + cediranib 30 mg once daily (OS analysis included additional 45 pts given 45mg)	Cediranib 30 mg once daily	126	10.5
			Carboplatin AUC 6 + paclitaxel 200 mg/m^2 every 21 days (6-8 cycles) + placebo once daily	Placebo once daily	125	10.1
43	AVAiL[71,72]	Treatment naïve nonsquamous stage IIIb/IV or recurrent NSCLC with ECOG PS 0-1 in predominantly Caucasian population	Cisplatin 80 mg/m^2 day 1 + gemcitabine 1,250 mg/m^2 days 1 and 8 + bevacizumab 15 mg/kg day 1 every 21	Bevacizumab 15 mg/kg every 21 days	351	13.4

			days (6 cycles)			
			Cisplatin 80 mg/m^2 day 1 + gemcitabine 1,250 mg/m^2 days 1 and 8 + bevacizumab 7.5 mg/kg day 1 every 21 days (6 cycles)	Bevacizumab 7.5 mg/kg every 21 days	345	13.6
			Cisplatin 80 mg/m^2 day 1 + gemcitabine 1,250 mg/m^2 days 1 and 8 + placebo day 1 every 21 days (6 cycles)	Placebo every 21 days	347	13.1
44	Soria et al.[73]	Treatment naïve stage IIIb/IV or recurrent squamous NSCLC and /or brain metastasis with ECOG PS 0-1	Carboplatin AUC 6 + paclitaxel 200 mg/m^2 day 1 + dulanermin 8 mg/kg once daily days 1-5 every 21 days (6 cycles)	Dulanermin 8 mg/kg once daily days 1-5 every 21 days	42	9.8
			Carboplatin AUC 6 + paclitaxel 200 mg/m^2 day 1 every 21 days (6 cycles)	No maintenance	41	10.1
		Treatment naïve stage IIIb/IV or recurrent nonsquamous NSCLC and no brain metastasis with ECOG PS 0-1	Carboplatin AUC 6 + paclitaxel 200 mg/m^2 + bevacizumab 15 mg/kg day 1 every 21 days (6 cycles)	Bevacizumab 15 mg/kg day 1 every 21 days	44	15.1
			Carboplatin AUC 6 + paclitaxel 200 mg/m^2 + bevacizumab 15 mg/kg day 1 + dulanermin 8 mg/kg once daily days 1-5 every 21 days (6 cycles)	Bevacizumab 15 mg/kg day 1 + dulanermin 8 mg/kg once daily days 1-5 every 21 days	43	13.9
			Carboplatin AUC 6 +	Bevacizumab 15 mg/kg day 1 +	43	14.3

			paclitaxel 200 mg/m^2 + bevacizumab 15 mg/kg day 1 + dulanermin 20 mg/kg once daily days 1-2 every 21 days (6 cycles)	dulanermin 20 mg/kg once daily days 1-2 every 21 days		
45	Lynch et al.[74]	Treatment naïve stage IIIb/IV NSCLC with ECOG PS 0-1 (used modified WHO PS criteria for PFS)	Carboplatin AUC 6 + paclitaxel 175 mg/m^2 day 1 every 21 days (6 cycles) + phased placebo day 1 every 21 days (2 cycles) followed by ipilimumab 10 mg/kg day 1 every 21 days (4 cycles)	Ipilimumab 10 mg/kg every 12 weeks	68	12.22
			Carboplatin AUC 6 + paclitaxel 175 mg/m^2 day 1 every 21 days (6 cycles) + concurrent ipilimumab 10 mg/kg day 1 every 21 days (4 cycles) followed by placebo day 1 every 21 days (2 cycles)	Ipilimumab 10 mg/kg every 12 weeks	70	9.69
			Carboplatin AUC 6 + paclitaxel 175 mg/m^2 + placebo day 1 every 21 days (6 cycles)	Placebo every 12 weeks	66	8.28
46	Belani et al.[75,76]	Treatment naïve nonsquamous stage IIIb/IV or recurrent NSCLC with ECOG PS 0-1	Cisplatin 75 mg/m^2 + pemetrexed 500 mg/m^2 day 1 every 21 days (6 cycles) + axitinib 5 mg twice daily every 21 days	Axitinib	55	17.0
			Cisplatin 75 mg/m^2 + pemetrexed	Axitinib	58	14.7

			500 mg/m^2 day 1 every 21 days (6 cycles) + axitinib 5 mg twice daily days 2-19 every 21 days up to 6 cycles (last cycle days 2-21)			
			Cisplatin 75 mg/m^2 + pemetrexed 500 mg/m^2 day 1 every 21 days (6 cycles)	No maintenance	57	15.9
47	Blumenschein et al.[77]	Treatment naïve nonsquamous stage IIIb/IV or recurrent NSCLC with ECOG PS 0-1 (included 2/186 PS 2 patients, 5/186 had previous chemotherapy noted as protocol violations)	Carboplatin AUC 6 + paclitaxel 200 mg/m^2 day 1 every 21 days (6 cycles) + motesanib 125 mg once daily	Motesanib 125 mg once daily	61	14.0
			Carboplatin AUC 6 + paclitaxel 200 mg/m^2 day 1 every 21 days (6 cycles) + motesanib 75 mg twice daily for 5 days followed by 2 treatment-free days weekly	Motesanib 75 mg twice daily for 5 days followed by 2 treatment-free days weekly	62	12.8
			Carboplatin AUC 6 + paclitaxel 200 mg/m^2 + bevacizumab 15mg/kg day 1 every 21 days (6 cycles)	Bevacizumab 15mg/kg every 21 days	63	14.0
48	BMS099[78]	Treatment naïve stage IIIb/IV or recurrent NSCLC with ECOG PS 0-1 (7/676 PS $\geq$2 pts were included)	Carboplatin AUC 6 + taxane (paclitaxel 225 mg/m^2 or docetaxel 75mg/m^2) day 1 every 21 days (6	Cetuximab 250 mg/m^2 weekly	338	9.69

			cycles) + cetuximab 400 mg/m^2 initial dose day 1, followed by 250 mg/m^2 weekly onwards			
			Carboplatin AUC 6 + taxane (paclitaxel 225 mg/m^2 or docetaxel 75mg/m^2) every 21 days (6 cycles)	No maintenance	338	8.38
49	CERTO[79,80]	Treatment naïve stage IIIb/IV NSCLC with ECOG PS 0-1, with later protocol amendment limiting to patients with EGFR expression $\geq$200	Platinum doublet ((cisplatin 80 mg/m^2 day 1 + vinorelbine 25 mg/m^2 days 1 and 8) or (cisplatin 75 mg/m^2 day 1 + gemcitabine 1250 mg/m^2 days 1 and 8)) every 21 days (6 cycles) + cilengitide 2000 mg once weekly + cetuximab 400 mg/m^2 initial dose day 1 followed by 250 mg/m^2 weekly onwards (cilengitide twice weekly arm closed at protocol amendment)	Cilengitide 2000 mg + cetuximab 250 mg/m^2 once weekly	85	13.6 (13.2 in EGFR $\geq$200)
			Platinum doublet ((cisplatin 80 mg/m^2 day 1 + vinorelbine 25 mg/m^2 days 1 and 8) or (cisplatin 75 mg/m^2 day 1 + gemcitabine	Cetuximab 250 mg/m^2 once weekly	84	9.7 (11.8 in EGFR $\geq$200)

			1250 mg/m^2 days 1 and 8)) every 21 days (6 cycles) + cetuximab 400 mg/m^2 initial dose day 1 followed by 250 mg/m^2 weekly onwards			
50	Hirsh et al.[81]	Treatment naïve stage IIIb/IV NSCLC with ECOG PS 0-1 (3/828 PS 2-3 included)	Carboplatin AUC 6 + paclitaxel 200 mg/m^2 day 1 + subcutaneous PF-3512676 0.2 mg/kg (maximum 20 mg/dose) days 8 and 15 every 21 days (6 cycles)	Subcutaneous PF-3512676 0.2 mg/kg weekly	408	10.0
			Carboplatin AUC 6 + paclitaxel 200 mg/m^2 every 21 days (6 cycles)	No maintenance	420	9.8
51	Manegold et al.[82]	Treatment naïve stage IIIb/IV NSCLC with ECOG PS 0-1	Cisplatin 75 mg/m^2 day 1 + gemcitabine 1250 mg/m^2 days 1 and 8 + subcutaneous PF-3512676 0.2 mg/kg days 8 and 15 every 21 days (6 cycles)	Subcutaneous PF-3512676 0.2 mg/kg weekly	416	11.0
			Cisplatin 75 mg/m^2 day 1 + gemcitabine 1250 mg/m^2 days 1 and 8 every 21 days (6 cycles)	No maintenance	423	10.7
52	SQUIRE[83–88]	Treatment naïve stage IV squamous NSCLC with ECOG PS 0-2 (1/1093 PS3 included)	Cisplatin 75 mg/m^2 day 1 + gemcitabine 1250 mg/m^2 days 1 and 8 every 21 days + necitumumab 800 mg days 1 and 8 every 21	Necitumumab 800 mg days 1 and 8 every 21 days	545	11.5

			days (6 cycles)			
			Cisplatin 75 mg/m^2 day 1 + gemcitabine 1250 mg/m^2 days 1 and 8 every 21 days (6 cycles)	No maintenance	548	9.9
53	SWOG S0819[89–91]	Treatment naïve stage IV or recurrent NSCLC with Zubrod PS 0-1	Carboplatin + paclitaxel (with or without bevacizumab) day 1 + cetuximab days 1, 8, and 15 every 21 days (6 cycles)	Cetuximab with (in bevacizumab appropriate population) or without bevacizumab	113 (in Bev inappropriate population (FISH positive))	11.2 (in Bev inappropriate population (FISH positive))
			Carboplatin + paclitaxel (with or without bevacizumab) day 1 every 21 days (6 cycles)	With (in bevacizumab appropriate population) or without bevacizumab	121 (in Bev inappropriate population (FISH positive))	8.7 (in Bev inappropriate population (FISH positive))
54	WJTOG 0203[92]	Chemonaïve stage IIIb/IV NSCLC with ECOG PS 0-1 in the Japanese population	Platinum doublet (3 cycles) followed by gefitinib 250 mg daily	Gefitinib 250 mg daily	298	13.7
			Platinum doublet (6 cycles)	No maintenance	297	12.9
55	H3E-AA-S110[93]	Chemonaïve stage IIIb/IV NSCLC with ECOG PS 0-1 who were non-smokers in the East Asian population	Cisplatin 75mg/m^2 + pemetrexed 500 mg/m^2 day 1 every 21 days (4 cycles)	Gefitinib 250 mg daily	39	-
			Cisplatin 75mg/m^2 + pemetrexed 500 mg/m^2 day 1 every 21 days (4 cycles)	Pemetrexed 500 mg/m^2 every 21 days (with optional cisplatin 75mg/m^2 every 21 days for first 2 cycles)	31	-
56	Karayama et al.[94]	Treatment naïve nonsquamous stage IIIb/IV NSCLC with ECOG PS 0-1	Carboplatin AUC 6 + pemetrexed 500 mg/m^2 + bevacizumab 15 mg/kg day 1 every 21	Pemetrexed 500 mg/m^2 + bevacizumab 15 mg/kg day 1 every 21 days	55	24.4

			days (4 cycles)			
			Carboplatin AUC 6 + pemetrexed 500 mg/m^2 + bevacizumab 15 mg/kg day 1 every 21 days (4 cycles)	Pemetrexed 500 mg/m^2 day 1 every 21 days	55	21.3

Sample size N and median OS represent values in the overall reported population of individual studies, and do not represent values for particular subgroups unless otherwise stated. [a]Maintenance treatments administered until disease progression, unacceptable toxicity, or decision to terminate by physician or patient, unless otherwise stated. [b]INTACT 1 and INTACT 2 overall study population included 2% and 3% stage IIIa NSCLC respectively. Hence, in our meta-analysis, we included reported results within PS 0-1. Median OS estimates were from the overall study population. IHC-immunohistochemical. [c]In EURTAC, nine subjects in the chemotherapy group continued chemotherapy beyond four cycles. [d]In TORCH, upon progression, patients were pre-assigned to cross-over treatments: patients in the erlotinib arm crossed-over to receive similar a regimen of cisplatin and gemcitabine while patients in the chemotherapy arm crossed-over to receive a similar erlotinib regimen. [e]In NEJ002, 3/228 ECOG PS 2 patients were included. [f]Included one EGFR wild-type and one ECOG PS 2. [f]Included 2/1159 ECOG PS 2. [g]Included 1/1079 ECOG PS 2.

Appendix Table 2: EGFR mutation positive OS and PFS estimates

	Study	First-line treatment/maintenance treatment		N	OS HR (95% CI)	PFS HR (95% CI)
		**Treatment**	**Reference**			
1	BEYOND[1,2]	Chemotherapy+bevacizumab/bevacizumab	Chemotherapy/no maintenance	40	0.90 (0.40-2.02)	0.27 (0.12-0.63)
2	INNOVATIONS[3]	Erlotinib+bevacizumab/ erlotinib+bevacizumab	Chemotherapy+bevacizumab/bevacizumab	32	0.45 (0.18-1.16)	0.91 (0.42-1.98)
3	EURTAC[4–7]	Erlotinib/erlotinib	Chemotherapy/no maintenance	174	0.92 (0.63-1.35)	0.34 (0.23-0.49)
4	TORCH[8]	Erlotinib/erlotinib	Chemotherapy/no maintenance	39	1.58 (0.70-3.57)	0.60 (0.30-1.20)
5	OPTIMAL[6,9–12]	Erlotinib/erlotinib	Chemotherapy/no maintenance	154	1.19 (0.83-1.71)	0.16 (0.11-0.26)
6	ENSURE[6,13–15]	Erlotinib/erlotinib	Chemotherapy/no maintenance	217	0.91 (0.63-1.31)	0.42 (0.27-0.66)
7	IPASS[16–18]	Gefitinib/gefitinib	Chemotherapy/no maintenance	261	1.00 (0.76-1.33)	0.48 (0.36-0.64)
8	NEJ002[6,19,20]	Gefitinib/gefitinib	Chemotherapy/no maintenance	228	0.89 (0.63-1.24)	0.32 (0.24-0.44)
9	WJTOG3405[6,21,22]	Gefitinib/gefitinib	Chemotherapy/no maintenance	172	1.25 (0.88-1.78)	0.49 (0.34-0.71)
10	First-SIGNAL[23]	Gefitinib/gefitinib	Chemotherapy/no maintenance	42	1.04 (0.50-2.18)	0.54 (0.27-1.10)
11	LUX-Lung 3 [6,24–26]	Afatinib/afatinib	Chemotherapy/no maintenance	345	0.88 (0.66-1.17)	0.58 (0.43-0.78)
12	LUX-Lung 6[6,25–27]	Afatinib/afatinib	Chemotherapy/no maintenance	364	0.93 (0.72-1.22)	0.28 (0.20-0.39)
13	TRIBUTE[29,30]	Chemotherapy+erlotinib/erlotinib	Chemotherapy/no maintenance	167	1.00 (0.69-1.45)	0.49 (0.20-1.20)
14	INTACT-1 & 2[31–33]	Chemotherapy+gefitinib/gefitinib	Chemotherapy/no maintenance	32	1.77 (0.50-6.23)	0.55 (0.19-1.60)
15	FASTACT-2[34]	Intercalated chemotherapy+erlotinib/erlotinib[a]	Chemotherapy/no maintenance	97	0.48 (0.27-0.84)	0.25 (0.16-0.39)
16	Yang et al.[35,36]	Chemotherapy/gefitinib	Gefitinib/gefitinib	50	1.57 (0.72-3.39)	0.83 (0.42-1.62)
17	JO25567[37,38]	Erlotinib+bevacizumab/ erlotinib+bevacizumab	Erlotinib/erlotinib	152	-	0.54 (0.36-0.79)
18	FLEX[39–41]	Chemotherapy+cetuximab/ cetuximab	Chemotherapy/no maintenance	133	1.22 (0.79-1.89)	0.70 (0.48-1.04)
19	Lux-Lung 7[28]	Afatinib/afatinib	Gefitinib/gefitinib	319	0.87 (0.66-1.15)	0.73 (0.57-0.95)
20	Cheng et al.[42]	Gefitinib+pemetrexed/ gefitinib+pemetrexed	Gefitinib/gefitinib	191	-	0.68 (0.48-0.96)

[a]Erlotinib 150 mg daily days 15-28 every 28 days, N-sample size

EGFR mutation subtypes deletion 19 and L858R

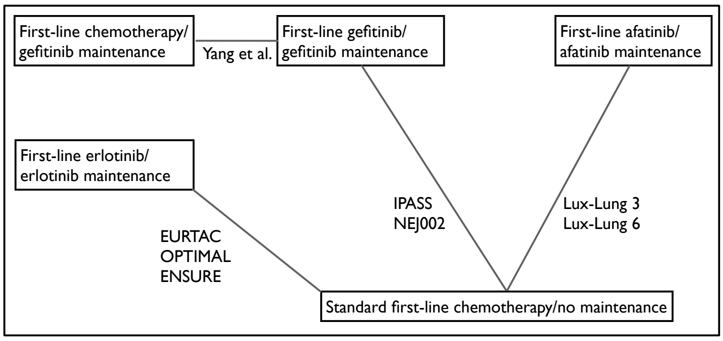

Appendix Figure 1: Network diagram of studies included in meta-analysis for OS in EGFR mutation subtypes deletion 19 and L858R NSCLC.

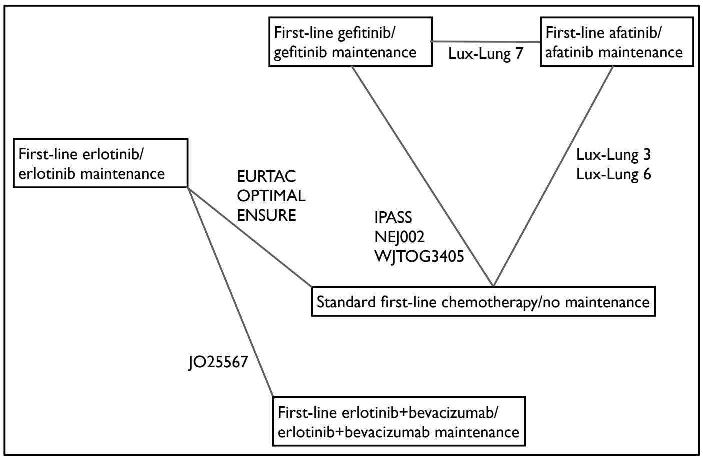

Appendix Figure 2: Network diagram of studies included in meta-analysis for PFS in EGFR mutation subtypes deletion 19 and L858R.

Appendix Table 3: EGFR mutation deletion 19 OS and PFS estimates

	Study	First-line treatment/maintenance treatment		N	OS HR (95% CI)	PFS HR (95% CI)
		**Treatment**	**Reference**			
1	EURTAC[4–7]	Erlotinib/erlotinib	Chemotherapy/no maintenance	115	0.94 (0.57-1.54)	0.27 (0.17-0.43)
2	OPTIMAL[6,9–12]	Erlotinib/erlotinib	Chemotherapy/no maintenance	82	1.52 (0.91-2.52)	0.13 (0.07-0.24)
3	ENSURE[6,13–15]	Erlotinib/erlotinib	Chemotherapy/no maintenance	118	0.79 (0.48-1.30)	0.20 (0.11-0.37)
4	IPASS[16–18]	Gefitinib/gefitinib	Chemotherapy/no maintenance	140	0.79 (0.54-1.15)	0.38 (0.26-0.56)
5	NEJ002[6,19,20]	Gefitinib/gefitinib	Chemotherapy/no maintenance	117	0.81 (0.51-1.31)	0.24 (0.15-0.38)
6	WJTOG3405[6,21,22]	Gefitinib/gefitinib	Chemotherapy/no maintenance	87	-	0.42 (0.26-0.66)
7	LUX-Lung 3[6,24–26]	Afatinib/afatinib	Chemotherapy/no maintenance	169	0.54 (0.36-0.79)	0.28 (0.18-0.44)
8	LUX-Lung 6[6,25–27]	Afatinib/afatinib	Chemotherapy/no maintenance	186	0.64 (0.44-0.94)	0.20 (0.13-0.32)
9	JO25567[37,38]	Erlotinib+bevacizumab/ erlotinib+bevacizumab	Erlotinib/erlotinib	80	-	0.41 (0.24-0.72)
10	Lux-Lung 7[28]	Afatinib/afatinib	Gefitinib/gefitinib	186	-	0.76 (0.55-1.06)
11	Yang et al.[35,36]	Chemotherapy/gefitinib	Gefitinib/gefitinib	26	2.36 (0.70-7.92)	-

Appendix Table 4: EGFR mutation L858R OS and PFS estimates

	Study	First-line treatment/maintenance treatment		N	OS HR (95% CI)	PFS HR (95% CI)
		**Treatment**	**Reference**			
1	EURTAC[4–7]	Erlotinib/erlotinib	Chemotherapy/no maintenance	59	0.99 (0.56-1.76)	0.53 (0.29-0.97)
2	OPTIMAL[6,9–12]	Erlotinib/erlotinib	Chemotherapy/no maintenance	72	0.92 (0.55-1.54)	0.26 (0.14-0.48)
3	ENSURE[6,13–15]	Erlotinib/erlotinib	Chemotherapy/no maintenance	98	1.05 (0.60-1.84)	0.57 (0.31-1.05)
4	IPASS[6–18]	Gefitinib/gefitinib	Chemotherapy/no maintenance	111	1.44 (0.90-2.30)	0.55 (0.35-0.87)
5	NEJ002[6,19,20]	Gefitinib/gefitinib	Chemotherapy/no maintenance	97	0.82 (0.49-1.38)	0.33 (0.20-0.54)
6	WJTOG3405[6,21,22]	Gefitinib/gefitinib	Chemotherapy/no maintenance	85	-	0.69 (0.44-1.07)
7	LUX-Lung 3[6,24–26]	Afatinib/afatinib	Chemotherapy/no maintenance	138	1.30 (0.80-2.11)	0.73 (0.46-1.16)
8	LUX-Lung 6[6,2]	Afatinib/afatinib	Chemotherapy/no maintenance	138	1.22 (0.81-1.83)	0.32 (0.19-0.54)
9	JO25567[37,38]	Erlotinib+bevacizumab/ erlotinib+bevacizumab	Erlotinib/erlotinib	72	-	0.67 (0.38-1.18)
10	Lux-Lung 7[28]	Afatinib/afatinib	Gefitinib/gefitinib	133	-	0.71 (0.48-1.06)
11	Yang et al. [35,36]	Chemotherapy/gefitinib	Gefitinib/gefitinib	24	1.23 (0.41-3.67)	-

Appendix Table 5: EGFR mutation wild-type OS and PFS estimates

	Study	First-line treatment/maintenance treatment		N	OS HR (95% CI)	PFS HR (95% CI)
		**Treatment**	**Reference**			
1	BEYOND[1,2]	Chemotherapy+bevacizumab/bevacizumab	Chemotherapy/no maintenance	111	0.57 (0.36-0.89)	0.33 (0.21-0.53)
2	TASK[43]	Erlotinib+bevacizumab/erlotinib+bevacizumab	Chemotherapy+bevacizumab/bevacizumab	59	-	2.07 (0.98-4.40)
3	INNOVATIONS[3]	Erlotinib+bevacizumab/erlotinib+bevacizumab	Chemotherapy+bevacizumab/bevacizumab	127	1.76 (1.13-2.74)	2.07 (1.42-3.02)
4	TORCH[8]	Erlotinib/erlotinib	Chemotherapy/no maintenance	236	1.29 (0.97-1.71)	2.07 (1.58-2.71)
5	IPASS[16–18]	Gefitinib/gefitinib	Chemotherapy/no maintenance	176	1.18 (0.86-1.63)	2.85 (2.05-3.98)
6	First-SIGNAL[23]	Gefitinib/gefitinib	Chemotherapy/no maintenance	54	1.00 (0.52-1.91)	1.42 (0.82-2.47)
7	TRIBUTE[29,30]	Chemotherapy+erlotinib/erlotinib	Chemotherapy/no maintenance	177	1.02 (0.71-1.46)	1.24 (0.92-1.66)
8	INTACT-1 & 2[31–33]	Chemotherapy+gefitinib/gefitinib	Chemotherapy/no maintenance	280	0.91 (0.67-1.23)	0.73 (0.53-1.01)
9	FASTACT-2[34]	Intercalated chemotherapy+erlotinib/erlotinib[a]	Chemotherapy/no maintenance	136	0.77 (0.53-1.11)	0.97 (0.69-1.36)
10	Yang et al. [35,36]	Chemotherapy/gefitinib	Gefitinib/gefitinib	24	0.62 (0.22-1.72)	0.18 (0.06-0.51)
11	FLEX[39–41]	Chemotherapy+cetuximab/cetuximab	Chemotherapy/no maintenance	838	0.91 (0.78-1.06)	1.02 (0.87-1.19)

[a]Erlotinib 150 mg daily days 15-28 every 28 days, N-sample size

Appendix Table 6: Nonsquamous OS and PFS estimates

	Study	First-line treatment/maintenance treatment		N	OS HR (95% CI)	PFS HR (95% CI)
		**Treatment**	**Reference**			
1	E4599[44]	Chemotherapy+bevacizumab/bevacizumab	Chemotherapy/no maintenance	850	0.79 (0.67-0.92)	0.66 (0.57-0.77)
2	PRONOUNCE[46,47]	Chemotherapy/pemetrexed	Chemotherapy+bevacizumab/bevacizumab	361	1.07 (0.83-1.36)	1.06 (0.84-1.35)
3	PointBreak[48,49]	Chemotherapy+bevacizumab/pemetrexed+bevacizumab	Chemotherapy+bevacizumab/bevacizumab	939	1.00 (0.86-1.16)	0.83 (0.71-0.96)
4	TASK[43]	Erlotinib+bevacizumab/ erlotinib+bevacizumab	Chemotherapy+bevacizumab/bevacizumab	124	1.24 (0.75-2.05)	2.05 (1.11-3.77)
5	INNOVATIONS[3]	Erlotinib+bevacizumab/ erlotinib+bevacizumab	Chemotherapy+bevacizumab/bevacizumab	224	1.41 (1.01-1.97)	1.85 (1.39-2.45)
6	TORCH[8]	Erlotinib/erlotinib	Chemotherapy/no	422	1.34 (1.06-1.69)	1.50 (1.22-
7	FLEX[39–41]	Chemotherapy+cetuximab/cetuximab	Chemotherapy/no maintenance	532	0.94 (0.77-1.15)	-
8	INSPIRE[50]	Chemotherapy+necitumumab/necitumumab	Chemotherapy/no maintenance	633	1.01 (0.84-1.21)	0.96 (0.80-1.16)
9	Paz-Ares et al.[51]	Chemotherapy+conatumumab/conatumumab	Chemotherapy/no maintenance	128	0.92 (0.59-1.44)	1.08 (0.74-1.57)
10	Reck et al.[52]	Chemotherapy+tigatumumab/tigatumumab	Chemotherapy/no maintenance	68	1.13 (0.62-2.05)	0.84 (0.48-1.47)
11	NVALT-4[53]	Chemotherapy+celecoxib/celecoxib	Chemotherapy/no maintenance	134	1.00 (0.69-1.45)	0.91 (0.65-1.27)
12	LUN06-116[54]	Chemotherapy+bevacizumab+enzastaurin/bevacizumab+enzastaurin	Chemotherapy+bevacizumab/bevacizumab	40	0.83 (0.31-2.22)	1.04 (0.49-2.21)
13	NVALT-12[55–58]	Chemotherapy+bevacizumab+nitroglycerin/bevacizumab+nitroglycerin	Chemotherapy+bevacizumab/bevacizumab	223	1.02 (0.71-1.46)	1.27 (0.96-1.67)
14	Lee et al.[59,60]	Chemotherapy+thalidomide/thalidomide	Chemotherapy/no maintenance	483	1.32 (1.10-1.60)	1.26 (1.05-1.52)
15	Langer et al.[61]	Chemotherapy+figitumumab/figitumumab	Chemotherapy/no maintenance	79	1.18 (0.71-1.96)	-
16	ATTRACT-1[62]	Chemotherapy+vadimezan/vadimezan	Chemotherapy/no maintenance	978	0.98 (0.80-1.19)	-
17	Scagliotti et al.[63]	Pemetrexed+pazopanib/pazopanib	Chemotherapy/no maintenance	97	1.22 (0.64-2.33)	0.75 (0.43-1.28)
18	Twelves et al. [64]	Chemotherapy+axitinib/axitinib	Chemotherapy+bevacizumab/bevacizumab	118	1.12 (0.74-1.69)	1.09 (0.68-1.76)
19	ESCAPE[65]	Chemotherapy+sorafenib[a]/sorafenib	Chemotherapy/no maintenance	703	0.98 (0.78-1.23)	0.91 (0.76-1.09)
20	NExUS[66]	Chemotherapy+sorafenib/sorafenib	Chemotherapy/no maintenance	772	0.98 (0.83-1.16)	0.83 (0.71-0.97)
21	MONET-1[67]	Chemotherapy+motesanib/motesanib	Chemotherapy/no maintenance	1090	0.90 (0.78-1.04)	0.79 (0.68-0.90)
22	BR.24[69,70]	Chemotherapy+cediranib/cediranib	Chemotherapy/no maintenance	134	-	0.89 (0.58-1.36)
23	AVAiL[b 71,72]	Chemotherapy+bevacizumab/bevacizumab	Chemotherapy/no maintenance	698	1.03 (0.86-1.23)	0.85 (0.73-1.00)
24	AVAiL[c 71,72]	Chemotherapy+bevacizumab/bevacizumab	Chemotherapy/no maintenance	692	0.93 (0.78-1.11)	0.75 (0.64-0.87)
25	INTACT-2[d 32,33]	Chemotherapy+gefitinib/gefitinib	Chemotherapy/no maintenance	380	0.97 (0.77-1.23)	-
26	INTACT-2[e 32,33]	Chemotherapy+gefitinib/gefitinib	Chemotherapy/no maintenance	371	0.87 (0.68-1.10)	-
27	Soria et al.[f]	Chemotherapy+bevacizumab+dulanermin/bevacizumab+dulanermin	Chemotherapy+bevacizumab/bevacizumab	87	1.03 (0.57-1.86)	0.83 (0.49-1.40)
28	Soria et al.[g]	Chemotherapy+bevacizumab+dulanermin/bevacizumab+dulanermin	Chemotherapy+bevacizumab/bevacizumab	87	1.04 (0.59-1.85)	0.88 (0.52-1.48)
29	Lynch et al.[h 74]	Chemotherapy+ipilimumab/ipilimumab	Chemotherapy/no maintenance	94	1.17 (0.74-1.86)	0.81 (0.53-1.26)
30	Lynch et al.[i 74]	Chemotherapy+ipilimumab/ipilimumab	Chemotherapy/no maintenance	96	0.96 (0.60-1.53)	0.88 (0.57-1.35)
31	Belani et al.[75,76]	Chemotherapy+axitinib/axitinib	Chemotherapy/no maintenance	112	1.05 (0.65-1.69)	0.89 (0.56-1.42)
32	Belani et al.[j 75,76]	Chemotherapy+axitinib/axitinib	Chemotherapy/no maintenance	115	1.45 (0.92-2.29)	1.02 (0.64-1.62)
33	Blumenschein et al.[k 77]	Chemotherapy+motesanib/motesanib	Chemotherapy+bevacizumab/bevacizumab	124	1.05 (0.67-1.63)	1.14 (0.73-1.76)
34	Blumenschein et al.[l 77]	Chemotherapy+motesanib/motesanib	Chemotherapy+bevacizumab/bevacizumab	125	1.18 (0.76-1.83)	1.22 (0.80-1.85)
35	BMS099[m 78]	Chemotherapy+cetuximab/cetuximab	Chemotherapy/no maintenance	354	0.88 (0.70-1.10)	0.90 (0.71-1.14)

36	BMS099[n 78]	Chemotherapy+cetuximab/cetuximab	Chemotherapy/no maintenance	190	0.95 (0.71-1.30)	-
37	CERTO[m 79,80]	Chemotherapy+cetuximab+cilengitide/cetuximab+cilengitide	Chemotherapy+cetuximab/cetuximab	111	0.94 (0.59-1.49)	0.84 (0.52-1.34)
38	CERTO[n 79,80]	Chemotherapy+cetuximab+cilengitide/cetuximab+cilengitide	Chemotherapy+cetuximab/cetuximab	17	0.46 (0.12-1.80)	0.87 (0.23-3.38)
39	Hirsh et al.[m 81]	Chemotherapy+toll-like-receptor PF-3512676/toll-like-receptor PF-3512676	Chemotherapy/no maintenance	478	0.97 (0.77-1.20)	-
40	Hirsh et al.[o 81]	Chemotherapy+toll-like-receptor PF-3512676/toll-like-receptor PF-3512676	Chemotherapy/no maintenance	51	0.81 (0.42-1.56)	-
41	Manegold et al.[m 82]	Chemotherapy+toll-like-receptor PF-3512676/toll-like-receptor PF-3512676	Chemotherapy/no maintenance	389	0.97 (0.75-1.24)	-
42	Manegold et al.[o 82]	Chemotherapy+toll-like-receptor PF-3512676/toll-like-receptor PF-3512676	Chemotherapy/no maintenance	57	0.77 (0.42-1.44)	-
43	Karayama et al.[94]	Chemotherapy+bevacizumab/pemetrexed+bevacizumab	Chemotherapy+bevacizumab/pemetrexed	110	0.87 (0.49-1.54)	0.73 (0.44-1.19)

[a]First-line sorafenib 400 mg twice daily days 2-19 every 21 days (6 cycles. [b]Bevacizumab 15 mg. [c]Bevacizumab 7.5 mg. [d]Gefitinib 500 mg. [e]Gefitinib 250 mg. [f]Dulanermin 8 mg/kg. [g]Dulanermin 20 mg/kg. [h]Phased ipilimumab. [i]Concurrent ipilimumab. [j]Axitinib 5 mg twice daily days 2-19 every 21 days up to 6 cycles (last cycle days 2-21). [k]Motesanib 125 mg once daily. [l]Motesanib 75 mg twice daily for 5 days followed by 2 treatment-free days weekly. [m]Adenocarcinoma histology. [n]Nonsquamous others histology. [o]Nonsquamous large cell histology. N-sample size.

Appendix Table 7: Squamous OS and PFS estimates

	**Study**	**First-line treatment/maintenance treatment**		**N**	**OS HR (95% CI)**	**PFS HR (95% CI)**
		Treatment	Reference			
1	TORCH[8]	Erlotinib/erlotinib	Chemotherapy/no maintenance	338	1.08 (0.84-1.39)	1.56 (1.24-1.95)
2	FLEX[39–41]	Chemotherapy+cetuximab/cetuximab	Chemotherapy/no maintenance	377	0.80 (0.64-1.00)	-
3	SQUIRE[83–88]	Chemotherapy+necitumumab/necitumumab	Chemotherapy/no maintenance	1093	0.84 (0.74-0.96)	0.85 (0.74-0.98)
4	Paz-Ares et al. [51]	Chemotherapy+conatumumab/conatumumab	Chemotherapy/no maintenance	44	0.51 (0.24-1.08)	0.47 (0.23-0.94)
5	Reck et al.[52]	Chemotherapy+tigatumumab/tigatumumab	Chemotherapy/no maintenance	29	1.19 (0.47-3.00)	0.90 (0.36-2.26)
6	NVALT-4[53]	Chemotherapy+celecoxib/celecoxib	Chemotherapy/no maintenance	42	0.56 (0.28-1.11)	0.56 (0.30-1.04)
7	Lee et al.[59,60]	Chemotherapy+thalidomide/thalidomide	Chemotherapy/no maintenance	239	0.84 (0.64-1.09)	0.84 (0.64-1.09)
8	Langer et al. [61]	Chemotherapy+figitumumab/figitumumab	Chemotherapy/no maintenance	602	1.16 (0.96-1.39)	-
9	ATTRACT-1[62]	Chemotherapy+vadimezan/vadimezan	Chemotherapy/no maintenance	265	1.10 (0.79-1.52)	-
10	ESCAPE[65]	Chemotherapy+sorafenib[a]/sorafenib	Chemotherapy/no maintenance	223	1.85 (1.22-2.81)	1.31 (0.94-1.83)
11	MONET-1[68]	Chemotherapy+motesanib/motesanib	Chemotherapy/no maintenance	360	0.89 (0.71-1.12)	0.85 (0.65-1.11)
12	BR.24[69,70]	Chemotherapy+cediranib/cediranib	Chemotherapy/no maintenance	56	-	0.66 (0.34-1.27)
13	Soria et al.[a 73]	Chemotherapy+dulanermin/dulanermin	Chemotherapy/no maintenance	83	0.88 (0.50-1.52)	1.12 (0.68-1.85)
14	BMS099[78]	Chemotherapy+cetuximab/cetuximab	Chemotherapy/no maintenance	132	0.85 (0.59-1.25)	0.70 (0.47-1.05)
15	CERTO[79,80]	Chemotherapy+cetuximab+cilengitide/cetuximab+cilengitide	Chemotherapy+cetuximab/cetuximab	41	0.68 (0.34-1.37)	0.52 (0.22-1.19)
16	Hirsh et al.[81]	Chemotherapy+toll-like-receptor PF-3512676/toll-like-receptor PF-3512676	Chemotherapy/no maintenance	199	0.96 (0.70-1.33)	-
17	Manegold et al.[82]	Chemotherapy+toll-like-receptor PF-3512676/toll-like-receptor PF-3512676	Chemotherapy/no maintenance	257	1.17 (0.88-1.57)	-
18	INTACT-2[b] [32,33]	Chemotherapy+gefitinib/gefitinib	Chemotherapy/no maintenance	304	1.36 (0.96-1.91)	-
19	INTACT-2[c] [32,33]	Chemotherapy+gefitinib/gefitinib	Chemotherapy/no maintenance	312	1.09 (0.76-1.56)	-
20	Lynch et al.[d] [74]	Chemotherapy+ipilimumab/ipilimumab	Chemotherapy/no maintenance	36	0.48 (0.22-1.03)	0.40 (0.18-0.87)
21	Lynch et al.[e] [74]	Chemotherapy+ipilimumab/ipilimumab	Chemotherapy/no maintenance	36	1.02 (0.50-2.08)	0.87 (0.42-1.81)

[a]Dulanermin 8 mg/kg. [b]Gefitinib 500 mg. [c]Gefitinib 250 mg. [d]Phased ipilimumab. [e]Concurrent ipilimumab. N-sample size

Appendix Table 8: FISH positive and unselected histology OS and PFS estimates

	Study	First-line treatment/maintenance treatment		N	OS HR (95% CI)	PFS HR (95% CI)
		Treatment	Reference			
1	BMS099[78]	Chemotherapy+cetuximab/cetuximab	Chemotherapy/no maintenance	54	1.92 (1.05-3.54)	1.54 (0.81-2.93)
2	S0819[89–91]	Chemotherapy+cetuximab/cetuximab	Chemotherapy/no maintenance	234	0.75 (0.57-1.00)	0.82 (0.63-1.07)

[a]Estimates from both bevacizumab appropriate (treated with bevacizumab) and inappropriate population (not treated with bevacizumab) in study S0819[89–91]

Appendix Table 9: FISH positive and squamous OS and PFS estimates

	Study	First-line treatment/maintenance treatment		N	OS HR (95% CI)	PFS HR (95% CI)
		Treatment	Reference			
1	SQUIRE[83–88]	Chemotherapy+necitumumab/necitumumab	Chemotherapy/no maintenance	208	0.70 (0.52-0.96)	0.71 (0.52-0.97)
2	S0819[89–91]	Chemotherapy+cetuximab/cetuximab (+bevacizumab/bevacizumab[a])	Chemotherapy/no maintenance (+bevacizumab/bevacizumab[a])	111	0.56 (0.37-0.84)	0.68 (0.46-1.01)

[a]Estimates from both bevacizumab appropriate (treated with bevacizumab) and inappropriate population (not treated with bevacizumab) in study S0819[89–91]

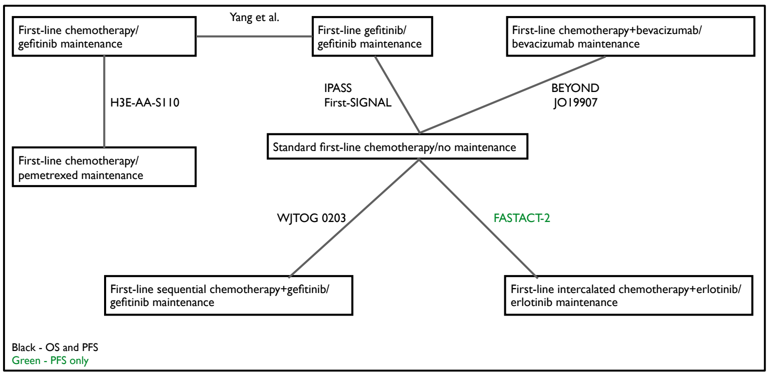

Appendix Figure 3: Network diagram of studies included in meta-analysis for OS and PFS in EGFR enriched and nonsquamous histology.

Appendix Table 10: Nonsquamous and EGFR-enriched OS and PFS estimates

	**Study**	**First-line treatment/maintenance treatment**		**N**	**OS HR (95% CI)**	**PFS HR (95% CI)**
		**Treatment**	**Reference**			
1	BEYOND[1,2]	Chemotherapy+bevacizumab/bevacizumab	Chemotherapy/no maintenance	276	0.68 (0.50-0.93)	0.40 (0.29-0.54)
2	JO19907[45]	Chemotherapy+bevacizumab/bevacizumab	Chemotherapy/no maintenance	175	0.99 (0.65-1.50)	0.61 (0.42-0.89)
3	IPASS[16–18]	Gefitinib/gefitinib	Chemotherapy/no maintenance	1217	0.90 (0.79-1.02)	0.74 (0.65-0.85)
4	First-SIGNAL[23]	Gefitinib/gefitinib	Chemotherapy/no maintenance	309	0.93 (0.72-1.21)	1.20 (0.94-1.52)
5	FASTACT-2[34]	Intercalated chemotherapy+erlotinib/erlotinib[a]	Chemotherapy/no maintenance	342	-	0.50 (0.40-0.63)
6	WJTOG 0203[92]	Sequential chemotherapy+gefitinib/gefitinib	Chemotherapy/no maintenance	467	0.79 (0.65-0.98)	0.60 (0.50-0.73)
7	Yang et al.[35,36]	Chemotherapy/gefitinib	Gefitinib/gefitinib	236	0.94 (0.68-1.31)	0.85 (0.63-1.13)
8	H3E-AA-S110[93]	Chemotherapy/gefitinib	Chemotherapy/pemetrexed	59	2.02 (0.70-5.82)	0.41 (0.19-0.88)

[a]Erlotinib 150 mg daily days 15-28 every 28 days

Appendix Table 11: Squamous and EGFR-enriched OS and PFS estimates

	**Study**	**First-line treatment/maintenance treatment**		**N**	**OS HR (95% CI)**	**PFS HR (95% CI)**
		**Treatment**	**Reference**			
1	FASTACT-2[34]	Intercalated chemotherapy+erlotinib/erlotinib[a]	Chemotherapy/no maintenance	109	-	0.89 (0.60-1.31)
2	WJTOG 0203[92]	Sequential chemotherapy+gefitinib/gefitinib	Chemotherapy/no maintenance	128	1.24 (0.85-1.79)	1.14 (0.80-1.62)

Additional results

Appendix Table 12: Overall survival and progression-free survival by EGFR FISH alone and EGFR FISH and histology

		Overall survival				Progression-free survival		
**First-line treatment**	**Maintenance treatment**	**SUCRA**	**HR (95% CrI)**	**Probability better than standard chemotherapy with no maintenance**	**Probability best**	**HR (95% CrI)**	**Probability better than standard chemotherapy with no maintenance**	**Probability best**
**EGFR FISH positive (unselected histology)**								
Chemotherapy+cetuximab [a]	Cetuximab	69.7%	0.92 (0.67-1.30)	0.70	0.70	0.92 (0.68-1.27)	0.70	0.70
Chemotherapy	No maintenance	30.3%	1.00	-	0.30	1.00	-	0.30
**EGFR FISH positive and squamous**								
Chemotherapy+cetuximab (+bevacizumab[b])	Cetuximab (+bevacizumab[b])	88.4%	0.56 (0.35-0.89)	0.99	0.78	0.68 (0.44-1.06)	0.96	0.56
Chemotherapy+necitumumab	Necitumumab	59.8%	0.70 (0.48-1.01)	0.97	0.22	0.71 (0.49-1.03)	0.96	0.44
Chemotherapy (+bevacizumab[b])	No maintenance (+bevacizumab[b])	1.8%	1.00	-	0.00	1.00	-	0.00

[a]Estimates from bevacizumab inappropriate (not treated with bevacizumab) population in study S0819[89–91]

[b]Estimates from both bevacizumab appropriate (treated with bevacizumab) and inappropriate (not treated with bevacizumab) population in study S0819[89–91]

Appendix Table 13: Overall survival and progression-free survival by histology in EGFR mutation-enriched populations

		Overall survival				Progression-free survival		
**First-line treatment**	**Maintenance treatment**	**SUCRA**	**HR (95% CrI)**	**Probability better than standard chemotherapy with no maintenance**	**Probability best**	**HR (95% CrI)**	**Probability better than standard chemotherapy with no maintenance**	**Probability best**
**EGFR enriched**								
**Nonsquamous**								
Chemotherapy	Pemetrexed	87.9%	0.42 (0.12-1.45)	0.92	0.80	1.88 (0.73-4.89)	0.10	0.00
Chemotherapy+bevacizumab	Bevacizumab	61.5%	0.78 (0.59-1.05)	0.95	0.09	0.48 (0.35-0.66)	1.00	0.50
Sequential chemotherapy+gefitinib	Gefitinib	60.5%	0.79 (0.60-1.05)	0.95	0.08	0.60 (0.43-0.84)	1.00	0.08
Chemotherapy	Gefitinib	45.0%	0.85 (0.56-1.31)	0.77	0.03	0.76 (0.48-1.22)	0.87	0.02
Gefitinib	Gefitinib	34.3%	0.91 (0.75-1.10)	0.86	0.00	0.90 (0.71-1.14)	0.82	0.00
Intercalated chemotherapy+erlotinib	Erlotinib[a]	-	-	-	-	0.50 (0.35-0.71)	1.00	0.39
Chemotherapy	No maintenance	10.8%	1.00	-	0.00	1.00	-	0.00
**Squamous**[b]								
Intercalated chemotherapy+erlotinib	Erlotinib[a]	-	-	-	-	0.89 (0.57-1.39)	0.70	0.65
Sequential chemotherapy+gefitinib	Gefitinib	-	-	-	-	1.14 (0.76-1.72)	0.26	0.13
Chemotherapy	No maintenance	-	-	-	-	1.00	-	0.22

[a]Erlotinib 150 mg daily days 15-28 every 28 days cycle

[b]No OS meta-estimates computed for squamous because there was only estimate from one study

Appendix Table 14: Progression-free survival by EGFR mutation status and subtypes

First-line treatment	Maintenance treatment	SUCRA	Progression-free survival, HR (95% CrI)	Probability better than standard chemotherapy with no maintenance (predictive)	Probability best
**EGFR mutation positive**					
Erlotinib+bevacizumab	Erlotinib+bevacizumab	94.0%	0.18 (0.11-0.30)	1.00 (1.00)	0.57
Chemotherapy+bevacizumab	Bevacizumab	81.0%	0.23 (0.12-0.45)	1.00 (1.00)	0.18
Intercalated chemotherapy+erlotinib	Erlotinib[a]	78.1%	0.25 (0.15-0.43)	1.00 (1.00)	0.13
Pemetrexed+gefitinib	Pemetrexed+gefitinib	65.1%	0.31 (0.19-0.51)	1.00 (1.00)	0.03
Erlotinib	Erlotinib	62.8%	0.32 (0.24-0.42)	1.00 (1.00)	0.00
Chemotherapy	Gefitinib	50.6%	0.38 (0.17-0.82)	0.99 (0.99)	0.03
Afatinib	Afatinib	49.0%	0.38 (0.29-0.49)	1.00 (1.00)	0.00
Chemotherapy+erlotinib	Erlotinib	36.0%	0.49 (0.18-1.31)	0.93 (0.92)	0.02
Gefitinib	Gefitinib	33.3%	0.45 (0.37-0.56)	1.00 (1.00)	0.00
Chemotherapy+gefitinib	Gefitinib	31.7%	0.55 (0.17-1.82)	0.85 (0.84)	0.03
Chemotherapy+cetuximab	Cetuximab	15.6%	0.70 (0.43-1.14)	0.93 (0.90)	0.00
Chemotherapy	No maintenance	2.8%	1.00	-	0.00
**EGFR mutation Deletion 19**					
Erlotinib+bevacizumab	Erlotinib+bevacizumab	99.9%	0.08 (0.04-0.17)	1.00 (1.00)	1.00
Erlotinib	Erlotinib	69.6%	0.20 (0.14-0.29)	1.00 (1.00)	0.00
Afatinib	Afatinib	54.8%	0.24 (0.18-0.32)	1.00 (1.00)	0.00
Gefitinib	Gefitinib	25.7%	0.33 (0.26-0.43)	1.00 (1.00)	0.00
Chemotherapy	No maintenance	0.0%	1.00	-	0.00
**EGFR mutation L858R**					
Erlotinib+bevacizumab	Erlotinib+bevacizumab	92.7%	0.29 (0.14-0.60)	1.00 (1.00)	0.82
Erlotinib	Erlotinib	62.8%	0.43 (0.29-0.63)	1.00 (1.00)	0.06
Afatinib	Afatinib	60.9%	0.45 (0.33-0.62)	1.00 (1.00)	0.11
Gefitinib	Gefitinib	33.7%	0.55 (0.42-0.72)	1.00 (1.00)	0.01
Chemotherapy	No maintenance	0.0%	1.00	-	0.00
**EGFR wild-type**					
Chemotherapy+bevacizumab	Bevacizumab	95.9%	0.33 (0.20-0.55)	1.00 (1.00)	0.64
Chemotherapy	Gefitinib	84.9%	0.42 (0.12-1.40)	0.93 (0.92)	0.36
Erlotinib+bevacizumab	Erlotinib+bevacizumab	70.3%	0.68 (0.36-1.29)	0.88 (0.87)	0.00
Chemotherapy+gefitinib	Gefitinib	70.1%	0.73 (0.49-1.08)	0.95 (0.92)	0.00
Intercalated chemotherapy+erlotinib	Erlotinib[a]	48.7%	0.97 (0.65-1.45)	0.56 (0.55)	0.00
Chemotherapy	No maintenance	45.9%	1.00	-	0.00
Chemotherapy+cetuximab	Cetuximab	43.7%	1.02 (0.78-1.34)	0.43 (0.44)	0.00
Chemotherapy+erlotinib	Erlotinib	28.7%	1.24 (0.86-1.79)	0.12 (0.15)	0.00
Erlotinib	Erlotinib	8.0%	2.07 (1.46-2.92)	0.00 (0.00)	0.00
Gefitinib	Gefitinib	3.6%	2.32 (1.65-3.24)	0.00 (0.00)	0.00

HR-hazard ratio, CrI-credible intervals

[a]Erlotinib 150 mg daily days 15-28 every 28 cycle

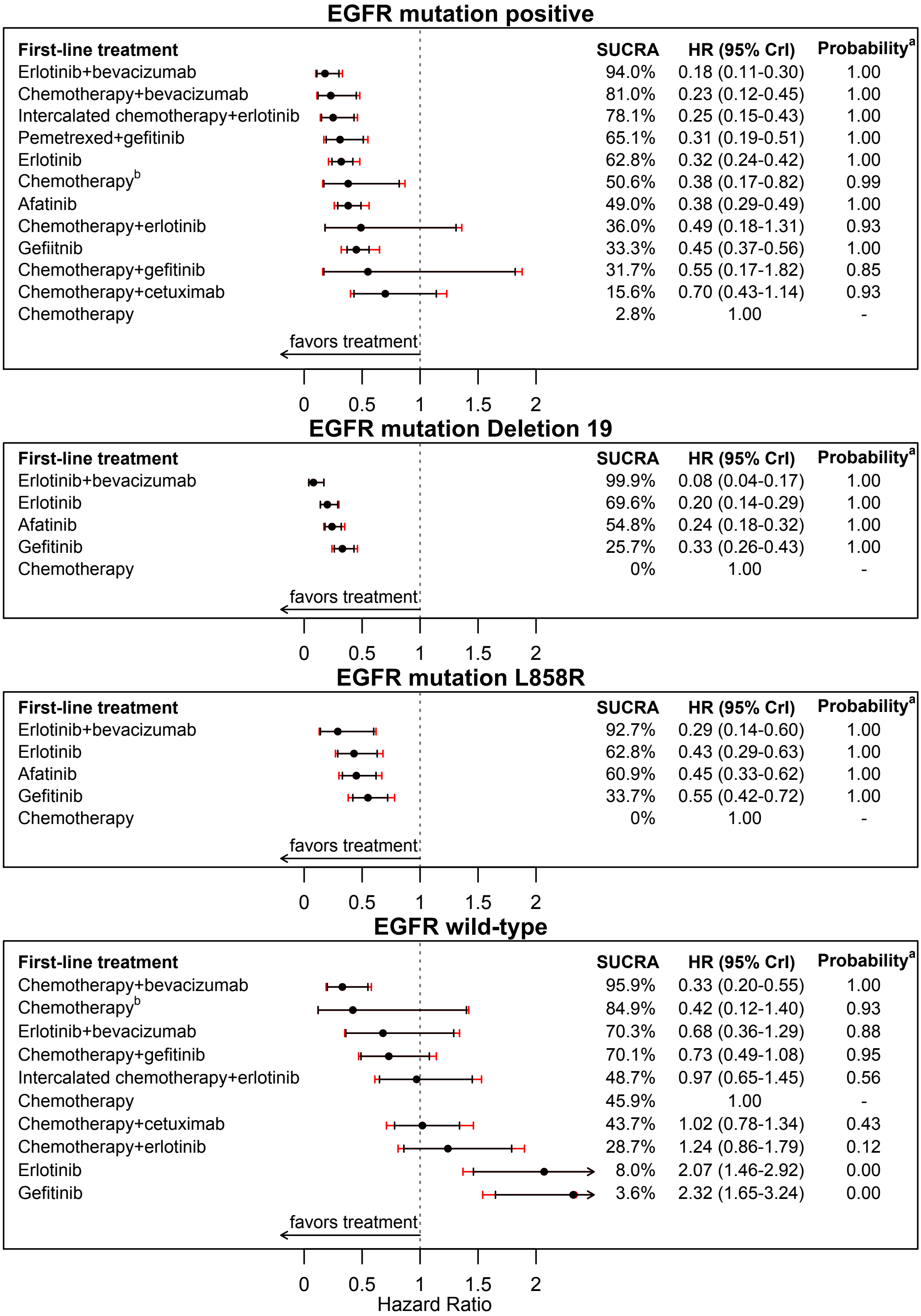

Appendix Figure 4: Progression-free survival hazard ratio (95% CrI), surface under the cumulative ranking curve (SUCRA) probability, and probability[a] better than standard chemotherapy with no maintenance by EGFR mutation status and subtypes for first-line therapies with maintenance regimens. [a]Probability better than standard chemotherapy with no maintenance. [b]First-line chemotherapy followed by gefitinib maintenance.

Appendix Table 15: Progression-free survival by histology

First-line treatment	Maintenance treatment	SUCRA	Progression-free survival, HR (95% CrI)	Probability better than standard chemotherapy with no maintenance	Probability best
**Nonsquamous**					
Chemotherapy+bevacizumab	Pemetrexed+bevacizumab	90.8%	0.61 (0.47-0.80)	1.00 (1.00)	0.18
Chemotherapy+bevacizumab+dulanermin	Bevacizumab+dulanermin	83.8%	0.63 (0.41-0.96)	0.98 (0.98)	0.16
Chemotherapy+bevacizumab	Bevacizumab	75.7%	0.74 (0.65-0.84)	1.00 (0.99)	0.00
Pemetrexed+pazopanib	Pazopanib	66.8%	0.75 (0.42-1.34)	0.84 (0.83)	0.13
Chemotherapy+cetuximab+cilengitide	Cetuximab+cilengitide	66.2%	0.76 (0.43-1.33)	0.84 (0.83)	0.11
Chemotherapy	Pemetrexed	65.6%	0.78 (0.57-1.08)	0.94 (0.92)	0.01
Chemotherapy+motesanib	Motesanib	62.4%	0.81 (0.68-0.98)	0.98 (0.95)	0.00
Chemotherapy+bevacizumab+enzastaurin	Bevacizumab+enzastaurin	62.0%	0.77 (0.34-1.73)	0.74 (0.74)	0.18
Chemotherapy+ipilimumab	Ipilimumab	56.1%	0.84 (0.60-1.19)	0.84 (0.82)	0.01
Chemotherapy+tigatuzumab	Tigatuzumab	55.5%	0.84 (0.46-1.53)	0.72 (0.71)	0.07
Chemotherapy+bevacizumab	Pemetrexed	55.2%	0.84 (0.46-1.53)	0.72 (0.71)	0.05
Chemotherapy+sorafenib	Sorafenib	53.1%	0.87 (0.73-1.03)	0.96 (0.90)	0.00
Chemotherapy+cediranib	Cediranib	49.6%	0.89 (0.56-1.41)	0.69 (0.68)	0.02
Chemotherapy+axitinib	Axitinib	47.5%	0.90 (0.67-1.21)	0.76 (0.73)	0.00
Chemotherapy+cetuximab	Cetuximab	47.4%	0.90 (0.67-1.20)	0.77 (0.75)	0.00
Chemotherapy+celecoxib	Celecoxib	46.8%	0.91 (0.62-1.33)	0.69 (0.68)	0.01
Chemotherapy+bevacizumab+nitroglycerin	Bevacizumab+nitroglycerin	42.2%	0.94 (0.66-1.33)	0.64 (0.63)	0.00
Chemotherapy+necitumumab	Necitumumab	38.7%	0.96 (0.75-1.24)	0.63 (0.62)	0.00
Chemotherapy	No maintenance	29.9%	1.00	-	0.00
Chemotherapy+conatumumab	Conatumumab	28.0%	1.08 (0.71-1.64)	0.36 (0.36	0.00
Chemotherapy+thalidomide	Thalidomide	12.4%	1.26 (0.98-1.62)	0.03 (0.06	0.00
Erlotinib+bevacizumab	Erlotinib+bevacizumab	7.5%	1.40 (1.01-1.93)	0.02 (0.03	0.00
Erlotinib	Erlotinib	4.1%	1.50 (1.15-1.96)	0.00 (0.01	0.00
**Squamous**					
Chemotherapy+cetuximab+cilengitide	Cetuximab+cilengitide	89.5%	0.36 (0.13-1.00)	0.98 (0.97)	0.53
Chemotherapy+conatumumab	Conatumumab	83.0%	0.47 (0.22-1.00)	0.98 (0.97)	0.23
Chemotherapy+celecoxib	Celecoxib	75.5%	0.56 (0.28-1.10)	0.96 (0.95)	0.10
Chemotherapy+ipilimumab	Ipilimumab	71.3%	0.61 (0.34-1.10)	0.95 (0.94)	0.05
Chemotherapy+cediranib	Cediranib	64.5%	0.66 (0.32-1.35)	0.88 (0.87)	0.05
Chemotherapy+cetuximab	Cetuximab	62.4%	0.70 (0.44-1.10)	0.94 (0.92)	0.00
Chemotherapy+thalidomide	Thalidomide	47.7%	0.84 (0.60-1.18)	0.85 (0.82)	0.00
Chemotherapy+necitumumab	Necitumumab	46.8%	0.85 (0.66-1.10)	0.91 (0.87)	0.00
Chemotherapy+motesanib	Motesanib	46.5%	0.85 (0.61-1.19)	0.84 (0.81)	0.00
Chemotherapy+tigatumumab	Tigatumumab	42.7%	0.90 (0.33-2.45)	0.58 (0.58)	0.03
Chemotherapy	No maintenance	28.6%	1.00	-	0.00
Chemotherapy+dulanermin[a]	Dulanermin[a]	24.3%	1.12 (0.65-1.94)	0.33 (0.34)	0.00
Chemotherapy+sorafenib	Sorafenib	12.8%	1.31 (0.88-1.94)	0.09 (0.11)	0.00
Erlotinib	Erlotinib	4.4%	1.56 (1.15-2.12)	0.00 (0.01)	0.00

HR-hazard ratio, CrI-credible intervals. [a]Dulanermin 8 mg/kg.

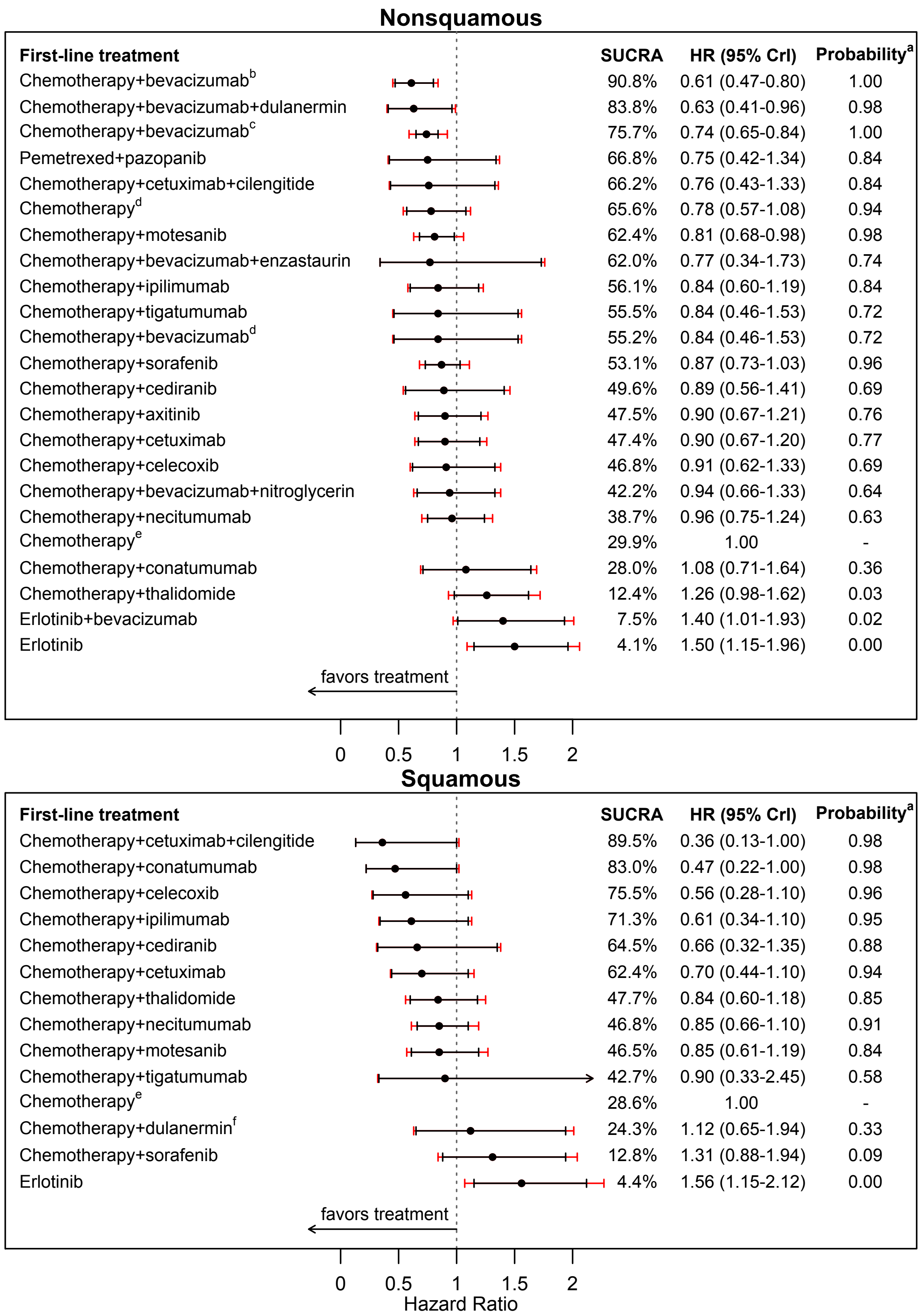

Appendix Figure 5: Progression-free survival hazard ratio (95% CrI), surface under the cumulative ranking curve (SUCRA) probability, and probability[a] better than standard chemotherapy with no maintenance by histology for first-line therapies with maintenance regimens. [a]Probability better than standard chemotherapy with no maintenance. [b]Pemetrexed+bevacizumab maintenance. [c]Bevacizumab maintenance. [d]Pemetrexed maintenance. [e]No maintenance. [f]Dulanermin 8 mg/kg.

Appendix Table 16: Progression-free survival by EGFR FISH alone and EGFR FISH and histology

First-line treatment	Maintenance treatment	SUCRA	Progression-free survival, HR (95% CrI)	Probability better than standard chemotherapy with no maintenance (predictive)	Probability best
**EGFR FISH positive (unselected histology)**					
Chemotherapy+cetuximab[a]	Cetuximab	70.2%	0.92 (0.68-1.27)	0.70 (0.68)	0.70
Chemotherapy	No maintenance	29.8%	1.00	-	0.30
**EGFR FISH positive and squamous**					
Chemotherapy+cetuximab (+bevacizumab[b])	Cetuximab (+bevacizumab[b])	75.8%	0.68 (0.44-1.06)	0.96 (0.94)	0.56
Chemotherapy+necitumumab	Necitumumab	70.2%	0.71 (0.49-1.03)	0.96 (0.95)	0.44
Chemotherapy (+bevacizumab[b])	No maintenance (+bevacizumab[b])	4.0%	1.00	-	0.00

[a]Estimates from bevacizumab inappropriate (not treated with bevacizumab) population in study S0819[89–91]
[b]Estimates from both bevacizumab appropriate (treated with bevacizumab) and inappropriate (not treated with bevacizumab) population in study S0819[89–91]

Head-to-head comparisons (EGFR mutation positive)

Appendix Table 17: Head-to-head comparisons of treatments for OS in EGFR mutation positive. HR estimates compare row treatments versus column treatments.

First-line treatment*	ICL	EB	A	CB	CE	E	G	CC	CG	CGmt
Intercalated chemotherapy+erlotinib (ICL)	1.00	1.19 (0.28-5.04)	0.53 (0.28-1.00)	0.53 (0.19-1.53)	0.48 (0.23-0.99)	0.46 (0.24-0.88)	0.47 (0.25-0.87)	0.39 (0.18-0.85)	0.27 (0.06-1.22)	0.30 (0.11-0.84)
Erlotinib+bevacizumab (EB)	0.84 (0.20-3.59)	1.00	0.45 (0.12-1.70)	0.45 (0.16-1.23)	0.40 (0.10-1.61)	0.39 (0.10-1.49)	0.39 (0.10-1.49)	0.33 (0.08-1.34)	0.23 (0.03-1.53)	0.25 (0.05-1.20)
Afatinib (A)	1.88 (1.00-3.54)	2.24 (0.59-8.46)	1.00	1.01 (0.41-2.44)	0.90 (0.58-1.42)	0.87 (0.65-1.17)	0.88 (0.70- 1.09)	0.74 (0.45-1.23)	0.51 (0.13-2.06)	0.56 (0.24-1.31)
Chemotherapy+bevacizumab (CB)	1.87 (0.65-5.36)	2.22 (0.81-6.08)	0.99 (0.41-2.42)	1.00	0.90 (0.35-2.33)	0.87 (0.35-2.12)	0.87 (0.36-2.11)	0.74 (0.28-1.97)	0.51 (0.10-2.57)	0.55 (0.17-1.86)
Chemotherapy+erlotinib (CE)	2.08 (1.01-4.30)	2.47 (0.62-9.81)	1.11 (0.70-1.74)	1.11 (0.43-2.89)	1.00	0.97 (0.61-1.54)	0.97 (0.62-1.51)	0.82 (0.44-1.53)	0.57 (0.13-2.38)	0.62 (0.24-1.58)
Erlotinib (E)	2.16 (1.13-4.10)	2.56 (0.67-9.74)	1.15 (0.85-1.54)	1.15 (0.47-2.82)	1.04 (0.65-1.65)	1.00	1.00 (0.75-1.34)	0.85 (0.50-1.43)	0.59 (0.14-2.37)	0.64 (0.27-1.54)
Gefitinib (G)	2.15 (1.15-4.02)	2.55 (0.67-9.63)	1.14 (0.92-1.42)	1.15 (0.47-2.78)	1.03 (0.66-1.61)	1.00 (0.75-1.33)	1.00	0.85 (0.51-1.40)	0.58 (0.14-2.34)	0.64 (0.28-1.46)
Chemotherapy+cetuximab (CC)	2.54 (1.18-5.45)	3.02 (0.74-12.19)	1.35 (0.81-2.25)	1.36 (0.51-3.63)	1.22 (0.65-2.27)	1.18 (0.70-1.98)	1.18 (0.71-1.96)	1.00	0.69 (0.16-2.97)	0.75 (0.29-1.98)
Chemotherapy+gefitinib (CG)	3.68 (0.82-16.54)	4.37 (0.65-29.39)	1.96 (0.49-7.88)	1.97 (0.39-9.98)	1.77 (0.42-7.45)	1.71 (0.42-6.92)	1.71 (0.43-6.90)	1.45 (0.34-6.21)	1.00	1.09 (0.22-5.50)
Chemotherapy (CGmt)	3.37 (1.20-9.49)	4.01 (0.84-19.10)	1.79 (0.76-4.22)	1.80 (0.54-6.02)	1.62 (0.63-4.13)	1.57 (0.65-3.75)	1.57 (0.69-3.59)	1.33 (0.50-3.49)	0.92 (0.18-4.61)	1.00

*with corresponding maintenance regimens as in Appendix Table 1
**corresponding rows and columns are reciprocals of each other.

Appendix Table 18: Head-to-head comparisons of treatments for PFS in EGFR mutation positive. HR estimates compare row treatments versus column treatments.

First-line treatment*	ICL	EB	A	CB	CE	E	G	CC	CG	CGmt	P[illegible]
Intercalated chemotherapy+erlotinib (ICL)	1.00	1.38 (0.66-2.89)	0.66 (0.36-1.19)	1.09 (0.46-2.57)	0.51 (0.17-1.56)	0.78 (0.43-1.43)	0.55 (0.31-0.98)	0.36 (0.17-0.73)	0.45 (0.12-1.69)	0.66 (0.26-1.71)	0.81 (0.3[illegible]
Erlotinib+bevacizumab (EB)	0.72 (0.35-1.52)	1.00	0.48 (0.27-0.84)	0.79 (0.41-1.52)	0.37 (0.12-1.12)	0.57 (0.36-0.90)	0.40 (0.23-0.70)	0.26 (0.13-0.52)	0.33 (0.09-1.21)	0.48 (0.19-1.22)	0.59 (0.2[illegible]
Afatinib (A)	1.52 (0.84-2.74)	2.09 (1.18-3.70)	1.00	1.65 (0.81-3.38)	0.77 (0.28-2.13)	1.19 (0.82-1.72)	0.84 (0.64-1.10)	0.54 (0.31-0.93)	0.69 (0.20-2.35)	1.01 (0.45-2.24)	1.23 (0.7[illegible]
Chemotherapy+bevacizumab (CB)	0.92 (0.39-2.16)	1.27 (0.66-2.46)	0.61 (0.30-1.24)	1.00	0.47 (0.14-1.53)	0.72 (0.37-1.41)	0.51 (0.25-1.02)	0.33 (0.14-0.75)	0.42 (0.11-1.65)	0.61 (0.22-1.71)	0.75 (0.3[illegible]
Chemotherapy+erlotinib (CE)	1.96 (0.64-5.98)	2.71 (0.90-8.17)	1.29 (0.47-3.56)	2.13 (0.65-7.00)	1.00	1.54 (0.55-4.25)	1.08 (0.40-2.94)	0.70 (0.23-2.09)	0.89 (0.19-4.17)	1.30 (0.37-4.54)	1.59 (0.5[illegible]
Erlotinib (E)	1.28 (0.70-2.33)	1.76 (1.12-2.78)	0.84 (0.58-1.22)	1.39 (0.71-2.73)	0.65 (0.24-1.80)	1.00	0.70 (0.50-1.00)	0.46 (0.26-0.80)	0.58 (0.17-1.98)	0.85 (0.37-1.94)	1.03 (0.5[illegible]
Gefitinib (G)	1.81 (1.02-3.23)	2.51 (1.44-4.36)	1.20 (0.91-1.57)	1.97 (0.98-4.00)	0.93 (0.34-2.52)	1.42 (1.00-2.01)	1.00	0.65 (0.38-1.10)	0.82 (0.24-2.78)	1.21 (0.57-2.56)	1.47 (0.9[illegible]
Chemotherapy+cetuximab (CC)	2.80 (1.36-5.77)	3.87 (1.91-7.83)	1.85 (1.07-3.19)	3.05 (1.33-6.99)	1.43 (0.48-4.27)	2.19 (1.26-3.82)	1.54 (0.91-2.62)	1.00	1.27 (0.35-4.64)	1.86 (0.74-4.67)	2.27 (1.1[illegible]
Chemotherapy+gefitinib (CG)	2.20 (0.59-8.16)	3.04 (0.83-11.17)	1.45 (0.43-4.93)	2.39 (0.61-9.44)	1.12 (0.24-5.23)	1.72 (0.50-5.89)	1.21 (0.36-4.09)	0.79 (0.22-2.85)	1.00	1.46 (0.35-6.09)	1.78 (0.4[illegible]
Chemotherapy (CGmt)	1.51 (0.58-3.88)	2.08 (0.82-5.29)	0.99 (0.45-2.21)	1.64 (0.59-4.58)	0.77 (0.22-2.68)	1.18 (0.52-2.69)	0.83 (0.39-1.76)	0.54 (0.21-1.35)	0.69 (0.16-2.85)	1.00	1.22 (0.5[illegible]
Pemetrexed+gefitinib (PG)	1.23 (0.59-2.57)	1.70 (0.83-3.48)	0.81 (0.48-1.38)	1.34 (0.58-3.10)	0.63 (0.21-1.89)	0.97 (0.55-1.71)	0.68 (0.43-1.07)	0.44 (0.22-0.88)	0.56 (0.15-2.05)	0.82 (0.34-1.97)	1.0[illegible]

*with corresponding maintenance regimens as in Appendix Table 1
**corresponding rows and columns are reciprocals of each other.

Head-to-head comparisons (Del 19)

Appendix Table 19: Head-to-head comparisons of treatments for OS in EGFR mutation Del 19. HR estimates compare row treatments versus column treatments.

First-line treatment*	A	G	E	CGmt
Afatinib (A)	1.00	0.74 (0.47-1.15)	0.57 (0.37-0.89)	0.31 (0.08-1.29)
Gefitinib (G)	1.36 (0.87-2.13)	1.00	0.77 (0.49-1.22)	0.42 (0.11-1.63)
Erlotinib (E)	1.75 (1.13-2.73)	1.29 (0.82-2.04)	1.00	0.55 (0.13-2.27)
Chemotherapy (CGmt)	3.20 (0.78-13.28)	2.36 (0.61-9.12)	1.83 (0.44-7.57)	1.00

*with corresponding maintenance regimens as in Appendix Table 1
**corresponding rows and columns are reciprocals of each other.

Appendix Table 20: Head-to-head comparisons of treatments for PFS in EGFR mutation Del 19. HR estimates compare row treatments versus column treatments.

First-line treatment*	A	G	E	EB
Afatinib (A)	1.00	0.73 (0.55 - 0.98)	1.20 (0.77 - 1.87)	2.93 (1.39 - 6.17)
Gefitinib (G)	1.37 (1.02 - 1.83)	1.00	1.64 (1.08 - 2.51)	4.00 (1.93 - 8.33)
Erlotinib (E)	0.83 (0.53 - 1.30)	0.61 (0.40 - 0.93)	1.00	2.44 (1.34 - 4.43)
Erlotinib+bevacizumab (EB)	0.34 (0.16 - 0.72)	0.25 (0.12 - 0.52)	0.41 (0.23 - 0.74)	1.00

*with corresponding maintenance regimens as in Appendix Table 1
**corresponding rows and columns are reciprocals of each other.

Head-to-head comparisons (L858R)

Appendix Table 21: Head-to-head comparisons of treatments for OS in EGFR mutation L858R. HR estimates compare row treatments versus column treatments.

First-line treatment*	A	G	E	CGmt
Afatinib (A)	1.00	1.12 (0.67-1.88)	1.28 (0.79-2.06)	0.91 (0.24 - 3.45)
Gefitinib (G)	0.89 (0.53-1.48)	1.00	1.13 (0.68-1.89)	0.81 (0.24 - 2.78)
Erlotinib (E)	0.78 (0.48-1.27)	0.88 (0.53-1.47)	1.00	0.72 (0.19 - 2.70)
Chemotherapy (CGmt)	1.09 (0.29-4.14)	1.23 (0.36-4.22)	1.40 (0.37-5.26)	1.00

*with corresponding maintenance regimens as in Appendix Table 1
**corresponding rows and columns are reciprocals of each other.

Appendix Table 22: Head-to-head comparisons of treatments for PFS in EGFR mutation L858R. HR estimates compare row treatments versus column treatments.

First-line treatment*	A	G	E	EB
Afatinib (A)	1.00	0.82 (0.59-1.16)	1.05 (0.63-1.73)	1.56 (0.70-3.48)
Gefitinib (G)	1.21 (0.86 - 1.70)	1.00	1.27 (0.79-2.03)	1.89 (0.86-4.14)
Erlotinib (E)	0.96 (0.58 - 1.58)	0.79 (0.49-1.26)	1.00	1.49 (0.80-2.79)
Erlotinib+bevacizumab (EB)	0.64 (0.29 - 1.43)	0.53 (0.24-1.16)	0.67 (0.36-1.25)	1.00

*with corresponding maintenance regimens as in Appendix Table 1
**corresponding rows and columns are reciprocals of each other.

Head-to-head comparisons (EGFR wild-type)

Appendix Table 23: Head-to-head comparisons of treatments for OS in EGFR wild-type. HR estimates compare row treatments versus column treatments.

First-line treatment*	CB	ICL	CGmt	CC	CG	EB	CE	G	E
Chemotherapy+bevacizumab (CB)	1.00	0.74 (0.38 - 1.42)	0.81 (0.22 - 2.96)	0.63 (0.36 - 1.10)	0.63 (0.34 - 1.16)	0.57 (0.35 - 0.93)	0.56 (0.29 - 1.07)	0.50 (0.28 - 0.91)	0.44 (0.24 - 0.81)
Intercalated chemotherapy+erlotinib (ICL)	1.35 (0.70 - 2.60)	1.00	1.09 (0.31 - 3.90)	0.85 (0.52 - 1.38)	0.85 (0.48 - 1.48)	0.77 (0.34 - 1.74)	0.76 (0.42 - 1.36)	0.68 (0.40 - 1.16)	0.60 (0.35 - 1.03)
Chemotherapy (CGmt)	1.24 (0.34 - 4.52)	0.91 (0.26 - 3.25)	1.00	0.77 (0.23 - 2.62)	0.77 (0.22 - 2.70)	0.70 (0.18 - 2.81)	0.69 (0.19 - 2.45)	0.62 (0.20 - 1.96)	0.55 (0.16 - 1.90)
Chemotherapy+cetuximab (CC)	1.60 (0.91 - 2.80)	1.18 (0.72 - 1.93)	1.29 (0.38 - 4.40)	1.00	1.00 (0.64 - 1.56)	0.91 (0.43 - 1.91)	0.89 (0.55 - 1.45)	0.80 (0.53 - 1.22)	0.71 (0.46 - 1.09)
Chemotherapy+gefitinib (CG)	1.60 (0.86 - 2.97)	1.18 (0.68 - 2.06)	1.29 (0.37 - 4.53)	1.00 (0.64 - 1.56)	1.00	0.91 (0.41 - 2.00)	0.89 (0.52 - 1.55)	0.80 (0.49 - 1.31)	0.71 (0.43 - 1.17)
Erlotinib+bevacizumab (EB)	1.76 (1.08 - 2.88)	1.30 (0.58 - 2.95)	1.43 (0.36 - 5.70)	1.10 (0.52 - 2.32)	1.10 (0.50 - 2.43)	1.00	0.98 (0.44 - 2.22)	0.88 (0.41 - 1.92)	0.78 (0.36 - 1.70)
Chemotherapy+erlotinib (CE)	1.79 (0.93 - 3.43)	1.32 (0.73 - 2.39)	1.45 (0.41 - 5.16)	1.12 (0.69 - 1.82)	1.12 (0.65 - 1.94)	1.02 (0.45 - 2.29)	1.00	0.90 (0.53 - 1.53)	0.79 (0.46 - 1.36)
Gefitinib (G)	1.99 (1.09 - 3.62)	1.48 (0.86 - 2.52)	1.61 (0.51 - 5.12)	1.25 (0.82 - 1.89)	1.25 (0.76 - 2.03)	1.13 (0.52 - 2.45)	1.11 (0.66 - 1.89)	1.00	0.88 (0.54 - 1.42)
Erlotinib (E)	2.27 (1.23 - 4.16)	1.68 (0.97 - 2.90)	1.83 (0.53 - 6.39)	1.42 (0.92 - 2.18)	1.42 (0.86 - 2.35)	1.29 (0.59 - 2.81)	1.27 (0.74 - 2.17)	1.14 (0.70 - 1.84)	1.00

*with corresponding maintenance regimens as in Appendix Table 1
**corresponding rows and columns are reciprocals of each other.

Appendix Table 24: Head-to-head comparisons of treatments for PFS in EGFR wild-type. HR estimates compare row treatments versus column treatments.

First-line treatment*	CB	ICL	CGmt	CC	CG	EB	CE	G	E
Chemotherapy+bevacizumab (CB)	1.00	0.34 (0.18-0.65)	0.79 (0.21-2.94)	0.32 (0.18-0.58)	0.45 (0.24-0.86)	0.48 (0.33-0.71)	0.27 (0.14-0.50)	0.14 (0.08-0.26)	0.16 (0.09-0.30)
Intercalated chemotherapy+erlotinib (ICL)	2.94 (1.53-5.66)	1.00	2.32 (0.65-8.31)	0.95 (0.59-1.54)	1.33 (0.76-2.33)	1.42 (0.67-3.03)	0.78 (0.45-1.35)	0.42 (0.25-0.71)	0.47 (0.28-0.80)
Chemotherapy (CGmt)	1.27 (0.34 - 4.71)	0.43 (0.12-1.54)	1.00	0.41 (0.12-1.41)	0.57 (0.16-2.03)	0.61 (0.16-2.40)	0.34 (0.10-1.19)	0.18 (0.06-0.58)	0.20 (0.06-0.71)
Chemotherapy+cetuximab (CC)	3.09 (1.73 - 5.52)	1.05 (0.65-1.71)	2.44 (0.71-8.43)	1.00	1.40 (0.87-2.24)	1.49 (0.75-2.99)	0.82 (0.52-1.29)	0.44 (0.29-0.68)	0.49 (0.32-0.76)
Chemotherapy+gefitinib (CG)	2.21 (1.16 - 4.22)	0.75 (0.43-1.32)	1.75 (0.49-6.22)	0.72 (0.45-1.15)	1.00	1.07 (0.51-2.26)	0.59 (0.35-1.00)	0.31 (0.19-0.53)	0.35 (0.21-0.59)
Erlotinib+bevacizumab (EB)	2.07 (1.42 - 3.03)	0.70 (0.33-1.50)	1.64 (0.42-6.41)	0.67 (0.33-1.34)	0.94 (0.44-1.98)	1.00	0.55 (0.26-1.15)	0.29 (0.14-0.61)	0.33 (0.16-0.68)
Chemotherapy+erlotinib (CE)	3.76 (2.00 - 7.07)	1.28 (0.74 - 2.21)	2.97 (0.84-10.5)	1.22 (0.77-1.91)	1.70 (1.00-2.90)	1.82 (0.87-3.80)	1.00	0.53 (0.33-0.88)	0.60 (0.36-0.99)
Gefitinib (G)	7.04 (3.79-12.98)	2.40 (1.41-4.02)	5.56 (1.73-17.75)	2.28 (1.47-3.47)	3.18 (1.89-5.29)	3.40 (1.64-6.97)	1.88 (1.13-3.06)	1.00	1.12 (0.69-1.81)
Erlotinib (E)	6.27 (3.38-11.65)	2.13 (1.26-3.63)	4.95 (1.41-17.46)	2.03 (1.31-3.15)	2.84 (1.69-4.77)	3.03 (1.47-6.27)	1.67 (1.01-2.76)	0.89 (0.55-1.46)	1.00

*with corresponding maintenance regimens as in Appendix Table 1
**corresponding rows and columns are reciprocals of each other.

References

1 Zhou C, Wu Y-L, Chen G, *et al.* BEYOND: A Randomized, Double-Blind, Placebo-Controlled, Multicenter, Phase III Study of First-Line Carboplatin/Paclitaxel Plus Bevacizumab or Placebo in Chinese Patients With Advanced or Recurrent Nonsquamous Non-Small-Cell Lung Cancer. *J Clin Oncol* 2015. DOI:10.1200/JCO.2014.59.4424.

2 Clinicaltrials.gov. A Study of Avastin (Bevacizumab) Versus Placebo in Combination With Carboplatin/Paclitaxel) in Patients With Advanced or Recurrent Non-Squamous Non-Small Cell Lung Cancer Who Have Not Received Previous Chemotherapy (NCT01364012). https://clinicaltrials.gov/ct2/show/record/NCT01364012 (accessed Aug 1, 2015).

3 Thomas M, Fischer J, Andreas S, *et al.* Erlotinib and bevacizumab versus cisplatin, gemcitabine and bevacizumab in unselected nonsquamous nonsmall cell lung cancer. *Eur Respir J* 2015; published online March 18. DOI:10.1183/09031936.00229014.

4 Rosell R, Carcereny E, Gervais R, *et al.* Erlotinib versus standard chemotherapy as first-line treatment for European patients with advanced EGFR mutation-positive non-small-cell lung cancer (EURTAC): a multicentre, open-label, randomised phase 3 trial. *Lancet Oncol* 2012; **13**: 239–46.

5 TARCEVA (erlotinib) tablets, for oral use [prescribing information] 2014. 2014. http://www.gene.com/download/pdf/tarceva_prescribing.pdf.

6 Lee CK, Wu Y-L, Ding PN, *et al.* Impact of Specific Epidermal Growth Factor Receptor (EGFR) Mutations and Clinical Characteristics on Outcomes After Treatment With EGFR Tyrosine Kinase Inhibitors Versus Chemotherapy in EGFR-Mutant Lung Cancer: A Meta-Analysis. *J Clin Oncol* 2015; published online April 20. DOI:10.1200/JCO.2014.58.1736.

7 Leon LF, Golsorkhi A, Liu S, Drozdowskyj A, Rosell R. 1273P Overall Survival Analyses Of First-Line Erlotinib Versus Chemotherapy In The EURTAC Study Population Controlling For The Use Of Post-Study Therapy. *Ann Oncol* 2014; **25**: iv447-iv448.

8 Gridelli C, Ciardiello F, Gallo C, *et al.* First-line erlotinib followed by second-line cisplatin-gemcitabine chemotherapy in advanced non-small-cell lung cancer: the TORCH randomized trial. *J Clin Oncol* 2012; **30**: 3002–11.

9 Zhou C, Wu Y-L, Chen G, *et al.* Erlotinib versus chemotherapy as first-line treatment for patients with advanced EGFR mutation-positive non-small-cell lung cancer (OPTIMAL, CTONG-0802): a multicentre, open-label, randomised, phase 3 study. *Lancet Oncol* 2011; **12**: 735–42.

10 Zhou C, Wu YL, Liu X, *et al.* Overall survival (OS) results from OPTIMAL (CTONG0802), a phase III trial of erlotinib (E) versus carboplatin plus gemcitabine (GC) as first-line treatment for Chinese patients with EGFR mutation-positive advanced non-small cell lung cancer (NSCLC). *ASCO Meet Abstr* 2012; **30**: 7520.

11 Chen G, Feng J, Zhou C, *et al.* Quality of life (QoL) analyses from optimal (CTONG-0802), a phase III, randomised, open-label study of first-line erlotinib versus chemotherapy in patients with advanced EGFR mutation-positive non-small-cell lung cancer (NSCLC). *Ann Oncol* 2013; **24**: 1615–22.

12 Zhou C, Wu YL, Chen G, *et al.* Final overall survival results from a randomised, Phase III study of erlotinib versus chemotherapy as first-line treatment of EGFR mutation-positive advanced non-small-cell lung cancer (OPTIMAL, CTONG-0802). *Ann Oncol* 2015; : mdv276-.

13 Wu Y-L, Liam C-K, Zhou C et al. First-line erlotinib versus cisplatin/gemcitabine (GP) in patients with advanced EGFR mutation-positive non-small-cell lung cancer (NSCLC): Interim analyses from the Phase 3, open-label, ENSURE study. *J Thorac Oncol* 2013; **8**: S603.

14 Lee C, Davies LC, Wu Y-L, *et al.* The impact on overall survival (OS) of first-line gefitinib (G) and erlotinib (E) and of clinical factors in advanced non-small cell lung cancer (NSCLC) with activating epidermal growth factor receptor mutations (EGFR mut) based on meta-analysis of 1,231 . In: J Clin Oncol 33, 2015 (suppl; abstr 8072). http://meetinglibrary.asco.org/content/144619-156.

15 Wu Y-L, Zhou C, Liam C-K, *et al.* First-line erlotinib versus gemcitabine/cisplatin in patients with advanced *EGFR* mutation-positive non-small-cell lung cancer: analyses from the phase III, randomized, open-label, ENSURE study. *Ann Oncol* 2015; : mdv270.

16 Mok TS, Wu Y, Thongprasert S, *et al.* Gefitinib or Carboplatin–Paclitaxel in Pulmonary Adenocarcinoma. *N Engl J Med* 2009; **361**: 947–57.

17 Fukuoka M, Wu Y-L, Thongprasert S, *et al.* Biomarker analyses and final overall survival results from a phase III, randomized, open-label, first-line study of gefitinib versus carboplatin/paclitaxel in clinically selected patients with advanced non-small-cell lung cancer

in Asia (IPASS). *J Clin Oncol* 2011; **29**: 2866–74.
18 Yang J, Wu Y., Saijo N, *et al.* Efficacy Outcomes in First-line Treatment of Advanced NSCLC With Gefitinib (G) vs Carboplatin/paclitaxel (C/P) by Epidermal Growth Factor Receptor (EGFR) Gene-copy Number Score and by Most Common EGFR Mutation Subtypes – Exploratory Data From IPASS. *Eur J Cancer 2011; 47 S633*.
19 Maemondo M, Inoue A, Kobayashi K, *et al.* Gefitinib or chemotherapy for non-small-cell lung cancer with mutated EGFR. *N Engl J Med* 2010; **362**: 2380–8.
20 Inoue a, Kobayashi K, Maemondo M, *et al.* Updated overall survival results from a randomized phase III trial comparing gefitinib with carboplatin-paclitaxel for chemo-naïve non-small cell lung cancer with sensitive EGFR gene mutations (NEJ002). *Ann Oncol* 2013; **24**: 54–9.
21 Mitsudomi T, Morita S, Yatabe Y, *et al.* Gefitinib versus cisplatin plus docetaxel in patients with non-small-cell lung cancer harbouring mutations of the epidermal growth factor receptor (WJTOG3405): an open label, randomised phase 3 trial. *Lancet Oncol* 2010; **11**: 121–8.
22 Yoshioka H, Mitsudomi T, Morita S, *et al.* Final overall survival results of WJTOG 3405, a randomized phase 3 trial comparing gefitinib (G) with cisplatin plus docetaxel (CD) as the first-line treatment for patients with non-small cell lung cancer (NSCLC) harboring mutations of the epidermal growt. In: J Clin Oncol 32:5s (suppl; abstr 8117). 2014.
23 Han J-Y, Park K, Kim S-W, *et al.* First-SIGNAL: first-line single-agent iressa versus gemcitabine and cisplatin trial in never-smokers with adenocarcinoma of the lung. *J Clin Oncol* 2012; **30**: 1122–8.
24 Sequist L V, Yang JC-H, Yamamoto N, *et al.* Phase III study of afatinib or cisplatin plus pemetrexed in patients with metastatic lung adenocarcinoma with EGFR mutations. *J Clin Oncol* 2013; **31**: 3327–34.
25 Yang JC, Wu Y, Schuler M, *et al.* Afatinib versus cisplatin-based chemotherapy for EGFR mutation-positive lung adenocarcinoma (LUX-Lung 3 and LUX-Lung 6): analysis of overall survival data from two randomised phase 3 trials. *Lancet Oncol* 2015; **2045**: 1–11.
26 Yang JC-H, Sequist L V., Schuler MH, *et al.* Overall survival (OS) in patients (pts) with advanced non-small cell lung cancer (NSCLC) harboring common (Del19/L858R) epidermal growth factor receptor mutations (EGFR mut): Pooled analysis of two large open-label phase III studies (LUX-Lung 3 [LL3] and. In: J Clin Oncol 32:5s (suppl; abstr 8004^). 2014.
27 Wu Y-L, Zhou C, Hu C-P, *et al.* Afatinib versus cisplatin plus gemcitabine for first-line treatment of Asian patients with advanced non-small-cell lung cancer harbouring EGFR mutations (LUX-Lung 6): an open-label, randomised phase 3 trial. *Lancet Oncol* 2014; **15**: 213–22.
28 Park K, Tan E, Zhang L, *et al.* Afatinib (A) vs gefitinib (G) as first-line treatment for patients (pts) with advanced non-small cell lung cancer (NSCLC) harboring activating EGFR mutations: LUX-Lung 7. *Ann Oncol* 2015; **26 (suppl_**.
29 Herbst RS, Prager D, Hermann R, *et al.* TRIBUTE: a phase III trial of erlotinib hydrochloride (OSI-774) combined with carboplatin and paclitaxel chemotherapy in advanced non-small-cell lung cancer. *J Clin Oncol* 2005; **23**: 5892–9.
30 Eberhard DA, Johnson BE, Amler LC, *et al.* Mutations in the epidermal growth factor receptor and in KRAS are predictive and prognostic indicators in patients with non-small-cell lung cancer treated with chemotherapy alone and in combination with erlotinib. *J Clin Oncol* 2005; **23**: 5900–9.
31 Giaccone G, Herbst RS, Manegold C, *et al.* Gefitinib in combination with gemcitabine and cisplatin in advanced non-small-cell lung cancer: a phase III trial--INTACT 1. *J Clin Oncol* 2004; **22**: 777–84.
32 Bell DW, Lynch TJ, Haserlat SM, *et al.* Epidermal growth factor receptor mutations and gene amplification in non-small-cell lung cancer: molecular analysis of the IDEAL/INTACT gefitinib trials. *J Clin Oncol* 2005; **23**: 8081–92.
33 Herbst RS, Giaccone G, Schiller JH, *et al.* Gefitinib in combination with paclitaxel and carboplatin in advanced non-small-cell lung cancer: a phase III trial--INTACT 2. *J Clin Oncol* 2004; **22**: 785–94.
34 Wu Y-L, Lee JS, Thongprasert S, *et al.* Intercalated combination of chemotherapy and erlotinib for patients with advanced stage non-small-cell lung cancer (FASTACT-2): a randomised, double-blind trial. *Lancet Oncol* 2013; **14**: 777–86.
35 Yang J, Kang J, Mok T. First-line pemetrexed plus cisplatin followed by gefitinib maintenance therapy versus gefitinib monotherapy in East Asian patients with locally advanced or metastatic non-squamous non-small cell lung cancer: A randomised, phase 3 trial. *Eur J Cancer* 2014;

2013: 2219–30.
36 Yang JC-H, Srimuninnimit V, Ahn M-J, *et al.* A randomized Phase 3 study comparing first-line pemetrexed plus cisplatin followed by gefitinib maintenance (PC/G) with gefitinib monotherapy (G) in East Asian patients (pts) with locally advanced or metastatic nonsquamous non-small cell lung cancer (nSqN. In: J Clin Oncol 33, 2015 (suppl; abstr 8041). http://meetinglibrary.asco.org/content/149032-156.
37 Seto T, Kato T, Nishio M, *et al.* Erlotinib alone or with bevacizumab as first-line therapy in patients with advanced non-squamous non-small-cell lung cancer harbouring EGFR mutations (JO25567): an open-label, randomised, multicentre, phase 2 study. *Lancet Oncol* 2014; **15**: 1236–44.
38 Kato T, Seto T, Nishio M, *et al.* Erlotinib plus bevacizumab (EB) versus erlotinib alone (E) as first-line treatment for advanced EGFR mutation–positive nonsquamous non-small cell lung cancer (NSCLC): An open-label randomized trial. In: J Clin Oncol 32:5s, 2014 (suppl; abstr 8005). .
39 Pirker R, Pereira JR, Szczesna A, *et al.* Cetuximab plus chemotherapy in patients with advanced non-small-cell lung cancer (FLEX): an open-label randomised phase III trial. *Lancet* 2009; **373**: 1525–31.
40 Pirker R, Pereira JR, von Pawel J, *et al.* EGFR expression as a predictor of survival for first-line chemotherapy plus cetuximab in patients with advanced non-small-cell lung cancer: analysis of data from the phase 3 FLEX study. *Lancet Oncol* 2012; **13**: 33–42.
41 Douillard J-Y, Pirker R, O'Byrne KJ, *et al.* Relationship Between EGFR Expression, EGFR Mutation Status, and the Efficacy of Chemotherapy Plus Cetuximab in FLEX Study Patients with Advanced Non–Small-Cell Lung Cancer. *J Thorac Oncol* 2014; **9**: 717–24.
42 Cheng Y, Murakami H, Yang P-C, *et al.* ORAL17.02 Randomized Trial of Gefitinib with and without Pemetrexed as First-Line Therapy in East-Asian Patients with Advanced NS NSCLC with EGFR Mutations. In: Journal of Thoracic Oncology, Volume 10, Number 9, Supplement 2, September 2015. S206.
43 Ciuleanu T, Tsai CM, Tsao CJ, *et al.* A phase II study of erlotinib in combination with bevacizumab versus chemotherapy plus bevacizumab in the first-line treatment of advanced non-squamous non-small cell lung cancer. *Lung Cancer* 2013; **82**: 276–81.
44 Sandler A, Gray R, Perry MC, *et al.* Paclitaxel-carboplatin alone or with bevacizumab for non-small-cell lung cancer. *N Engl J Med* 2006; **355**: 2542–50.
45 Niho S, Kunitoh H, Nokihara H, *et al.* Randomized phase II study of first-line carboplatin-paclitaxel with or without bevacizumab in Japanese patients with advanced non-squamous non-small-cell lung cancer. *Lung Cancer* 2012; **76**: 362–7.
46 Zinner RG, Obasaju CK, Spigel DR, *et al.* PRONOUNCE: Randomized, Open-Label, Phase III Study of First-Line Pemetrexed + Carboplatin Followed by Maintenance Pemetrexed versus Paclitaxel + Carboplatin + Bevacizumab Followed by Maintenance Bevacizumab in Patients ith Advanced Nonsquamous Non-Small-C. *J Thorac Oncol* 2015; **10**: 134–42.
47 Study of Participants With Advanced Non-Small Cell Lung Cancer (NCT00948675). ClinicalTrials.gov. https://clinicaltrials.gov/ct2/show/NCT00948675?term=NCT00948675&rank=1 (accessed Aug 2, 2015).
48 Patel JD, Socinski M a, Garon EB, *et al.* PointBreak: a randomized phase III study of pemetrexed plus carboplatin and bevacizumab followed by maintenance pemetrexed and bevacizumab versus paclitaxel plus carboplatin and bevacizumab followed by maintenance bevacizumab in patients with stage IIIB o. *J Clin Oncol* 2013; **31**: 4349–57.
49 Clinicaltrials.gov. A Study of Pemetrexed, Carboplatin and Bevacizumab in Patients With Nonsquamous Non-Small Cell Lung Cancer (NCT00762034). https://clinicaltrials.gov/ct2/show/NCT00762034 (accessed Aug 2, 2015).
50 Paz-ares L, Mezger J, Ciuleanu TE, *et al.* Necitumumab plus pemetrexed and cisplatin as first-line therapy in patients with stage IV non-squamous non-small-cell lung cancer (INSPIRE): an open-label , randomised, controlled phase 3 study. *Lancet Oncol* 2015; **16**: 328–37.
51 Paz-Ares L, Bálint B, de Boer RH, *et al.* A randomized phase 2 study of paclitaxel and carboplatin with or without conatumumab for first-line treatment of advanced non-small-cell lung cancer. *J Thorac Oncol* 2013; **8**: 329–37.
52 Reck M, Krzakowski M, Chmielowska E, *et al.* A randomized , double-blind , placebo-controlled phase 2 study of tigatuzumab (CS-1008) in combination with carboplatin / paclitaxel in patients with chemotherapy-naïve metastatic / unresectable non-small cell lung cancer. *Lung Cancer* 2013; **82**: 441–8.

53 Groen HJM, Sietsma H, Vincent A, *et al.* Randomized, placebo-controlled phase III study of docetaxel plus carboplatin with celecoxib and cyclooxygenase-2 expression as a biomarker for patients with advanced non-small-cell lung cancer: The NVALT-4 study. *J Clin Oncol* 2011; **29**: 4320–6.

54 Casey EM, Harb W, Bradford D, *et al.* Randomized, Double-Blinded, Multicenter, Phase II Study of Pemetrexed, Carboplatin, and Bevacizumab with Enzastaurin or Placebo in Chemonaïve Patients with Stage IIIB/IV Non-small Cell Lung Cancer. Hoosier Oncology Group LUN06-116. *J Thorac Oncol* 2010; **5**: 1815–20.

55 Dingemans A-MC, Groen HJM, Herder J, *et al.* A randomized phase II study of paclitaxel-carboplatin-bevacizumab (PCB) with or without nitroglycerin patches (NTG) in patients (pts) with stage IV nonsquamous non-small cell lung cancer (NSCLC): Nvalt 12 (NCT01171170). In: J Clin Oncol 32:5s, (suppl; abstr 8089). 2014.

56 Dingemans AC, Groen HJM, Herder G, *et al.* 1253PA RANDOMIZED PHASE II STUDY OF PACLITAXEL-CARBOPLATIN-BEVACIZUMAB (PCB) WITH OR WITHOUT NITROGLYCERIN PATCHES (NTG) IN PATIENTS (PTS) WITH STAGE IV NON-SQUAMOUS-NON-SMALL CELL LUNG CANCER (NS-NSCLC)(NVALT 12), IMPACT OF CIRCULATING VASCULAR ENDOTHELI. *Ann Oncol* 2014; **25**: iv440-iv440.

57 Clinicaltrials.gov. Paclitaxel-Carboplatin-Bevacizumab +/- Nitroglycerin in Metastatic Non-Squamous-Non-Small Cell Lung Cancer (NVALT12) NCT01171170. ClinicalTrials.gov. https://clinicaltrials.gov/ct2/show/record/NCT01171170 (accessed June 26, 2015).

58 Dingemans a.-MC, Groen HJM, Herder GJM, *et al.* A randomized phase II study comparing paclitaxel–carboplatin–bevacizumab with or without nitroglycerin patches in patients with stage IV nonsquamous nonsmall-cell lung cancer: NVALT12 (NCT01171170). *Ann Oncol* 2015; **26**: 2286–93.

59 Lee SM, Rudd R, Woll PJ, *et al.* Randomized double-blind placebo-controlled trial of thalidomide in combination with gemcitabine and Carboplatin in advanced non-small-cell lung cancer. *J Clin Oncol* 2009; **27**: 5248–54.

60 Sm L, Hackshaw a. A potential new enriching trial design for selecting non-small-cell lung cancer patients with no predictive biomarker for trials based on both histology and early tumor response: further analysis of a thalidomide trial. *Cancer Med* 2013; **2**: 360–6.

61 Langer CJ, Novello S, Park K, *et al.* Randomized, Phase III Trial of First-Line Figitumumab in Combination With Paclitaxel and Carboplatin Versus Paclitaxel and Carboplatin Alone in Patients With Advanced Non-Small-Cell Lung Cancer. *J Clin Oncol* 2014; **32**: 1–10.

62 Lara PN, Douillard J-Y, Nakagawa K, *et al.* Randomized phase III placebo-controlled trial of carboplatin and paclitaxel with or without the vascular disrupting agent vadimezan (ASA404) in advanced non-small-cell lung cancer. *J Clin Oncol* 2011; **29**: 2965–71.

63 Scagliotti G V, Felip E, Besse B, *et al.* An open-label, multicenter, randomized, phase II study of pazopanib in combination with pemetrexed in first-line treatment of patients with advanced-stage non-small-cell lung cancer. *J Thorac Oncol* 2013; **8**: 1529–37.

64 Twelves C, Chmielowska E, Havel L, *et al.* Randomised phase II study of axitinib or bevacizumab combined with paclitaxel/carboplatin as first-line therapy for patients with advanced non-small-cell lung cancer. *Ann Oncol* 2014; **25**: 132–8.

65 Scagliotti G, Novello S, von Pawel J, *et al.* Phase III study of carboplatin and paclitaxel alone or with sorafenib in advanced non-small-cell lung cancer. *J Clin Oncol* 2010; **28**: 1835–42.

66 Paz-Ares LG, Biesma B, Heigener D, *et al.* Phase III, randomized, double-blind, placebo-controlled trial of gemcitabine/cisplatin alone or with sorafenib for the first-line treatment of advanced, nonsquamous non-small-cell lung cancer. *J Clin Oncol* 2012; **30**: 3084–92.

67 Scagliotti G V., Vynnychenko I, Park K, *et al.* International, randomized, placebo-controlled, double-blindphase III study of motesanib plus carboplatin/paclitaxel in patients with advanced nonsquamous non-small-cell lung cancer: MONET1. *J Clin Oncol* 2012; **30**: 2829–36.

68 Novello S, Giorgio V, Vynnychenko I, *et al.* Motesanib Plus Carboplatin/Paclitaxel in Patients With Advanced Squamous Non–Small-Cell Lung Cancer: Results From the Randomized Controlled MONET1 Study. *J Thorac Oncol* 2014; : 1154–61.

69 Goss GD, Arnold A, Shepherd F, *et al.* Randomized, double-blind trial of carboplatin and paclitaxel with either daily oral cediranib or placebo in advanced non-small-cell lung cancer: NCIC clinical trials group BR24 study. *J Clin Oncol* 2010; **28**: 49–55.

70 Clinicaltrials.gov. Paclitaxel and Carboplatin With or Without Cediranib Maleate in Treating Patients With Stage III or Stage IV Non-Small Cell Lung Cancer: NCT00245154. https://clinicaltrials.gov/ct2/show/record/NCT00245154?term=cediranib+nsclc+br24&rank=1 (accessed July 9, 2015).

71 Reck M, von Pawel J, Zatloukal P, *et al.* Phase III trial of cisplatin plus gemcitabine with either placebo or bevacizumab as first-line therapy for nonsquamous non-small-cell lung cancer: AVAil. *J Clin Oncol* 2009; **27**: 1227–34.
72 Reck M, von Pawel J, Zatloukal P, *et al.* Overall survival with cisplatin-gemcitabine and bevacizumab or placebo as first-line therapy for nonsquamous non-small-cell lung cancer: results from a randomised phase III trial (AVAiL). *Ann Oncol* 2010; **21**: 1804–9.
73 Soria J, Ma Z, Zatloukal P, *et al.* Randomized Phase II Study of Dulanermin in Combination With Paclitaxel , Carboplatin , and Bevacizumab in Advanced Non – Small-Cell Lung Cancer. *J Clin Oncol* 2011; **29**. DOI:10.1200/JCO.2011.37.2623.
74 Lynch TJ, Bondarenko I, Luft A, *et al.* Ipilimumab in combination with paclitaxel and carboplatin as first-line treatment in stage IIIB/IV non-small-cell lung cancer: results from a randomized, double-blind, multicenter phase II study. *J Clin Oncol* 2012; **30**: 2046–54.
75 Belani CP, Yamamoto N, Bondarenko IM, *et al.* Randomized phase II study of pemetrexed/cisplatin with or without axitinib for non-squamous non-small-cell lung cancer. *BMC Cancer* 2014; **14**: 290.
76 Evaluation Of The Efficacy Of The Combination Of Axitinib With Pemetrexed And Cisplatin In The Treatment Of Non-Squamous Non-Small Cell Lung Cancer. ClinicalTrials.gov. https://clinicaltrials.gov/ct2/show/NCT00768755 (accessed July 3, 2015).
77 Blumenschein GR, Kabbinavar F, Menon H, *et al.* A phase II, multicenter, open-label randomized study of motesanib or bevacizumab in combination with paclitaxel and carboplatin for advanced nonsquamous non-small-cell lung cancer. *Ann Oncol* 2011; **22**: 2057–67.
78 Lynch TJ, Patel T, Dreisbach L, *et al.* Cetuximab and first-line taxane/carboplatin chemotherapy in advanced non-small-cell lung cancer: results of the randomized multicenter phase III trial BMS099. *J Clin Oncol* 2010; **28**: 911–7.
79 Vansteenkiste JF, Barlesi F, Waller C, *et al.* 1235PCILENGITIDE (CIL) COMBINED WITH CETUXIMAB AND PLATINUM-BASED CHEMOTHERAPY AS FIRST-LINE TREATMENT IN ADVANCED NON-SMALL CELL LUNG CANCER (NSCLC) PATIENTS (PTS): PHASE II RANDOMISED CERTO STUDY. *Ann Oncol* 2014; **25**: iv432-iv432.
80 Vansteenkiste J, Barlesi F, Waller CF, *et al.* Cilengitide combined with cetuximab and platinum-based chemotherapy as first-line treatment in advanced non-small-cell lung cancer (NSCLC) patients: results of an open-label, randomized, controlled phase II study (CERTO). *Ann Oncol* 2015; : 1–7.
81 Hirsh V, Paz-Ares L, Boyer M, *et al.* Randomized phase III trial of paclitaxel/carboplatin with or without PF-3512676 (toll-like receptor 9 agonist) as first-line treatment for advanced non-small-cell lung cancer. *J Clin Oncol* 2011; **29**: 2667–74.
82 Manegold C, van Zandwijk N, Szczesna a, *et al.* A phase III randomized study of gemcitabine and cisplatin with or without PF-3512676 (TLR9 agonist) as first-line treatment of advanced non-small-cell lung cancer. *Ann Oncol* 2012; **23**: 72–7.
83 Socinski MA, Paz-Ares L, Luft AV, *et al.* SQUIRE: A Randomized, Multicenter, Open-Label, Phase III Study of Gemcitabine-Cisplatin (GC) Chemotherapy Plus Necitumumab (Imc-11f8/ Ly3012211) Vs GC Alone In The First-Line Treatment Of Patients (Pts) With Stage IV Squamous Non-Small Cell Lung Cancer (S. In: Annals of Oncology 25 (Supplement 4): iv426–iv470. 2014.
84 Thatcher N, Hirsch FR, Szczesna A, *et al.* A randomized, multicenter, open-label, phase III study of gemcitabine-cisplatin (GC) chemotherapy plus necitumumab (IMC-11F8/LY3012211) versus GC alone in the first-line treatment of patients (pts) with stage IV squamous non-small cell lung cancer (sq-NSC. In: J Clin Oncol 32:5s, (suppl; abstr 8008^). 2014.
85 Socinski MA, Luft A, Szczesna A, *et al.* Subgroup analyses by performance status (PS) in the phase III SQUIRE study: First-line necitumumab (N) plus gemcitabine-cisplatin (GC) vs. GC in squamous non-small cell lung cancer (NSCLC). In: J Clin Oncol 33, (suppl; abstr e19023). 2015.
86 Thatcher N, Hirsch FR, Luft A V, *et al.* Necitumumab plus gemcitabine and cisplatin versus gemcitabine and cisplatin alone as first-line therapy in patients with stage IV squamous non-small-cell lung cancer (SQUIRE): an open-label, randomised, controlled phase 3 trial. *Lancet Oncol* 2015; **16**: 763–74.
87 Hirsch FR, Boyle TA, Thatcher N, *et al.* 32.05 EGFR IHC and FISH Correlative Analyses (SQUIRE Trial): Necitumumab + Gemcitabine-Cisplatin vs Gemcitabine-Cisplatin in 1st-Line Squamous NSCLC. In: WCLC 2015. .
88 Hirsch FR, Boyle TA, Thatcher N, *et al.* ORAL32.05 EGFR IHC and FISH Correlative

Analyses (SQUIRE Trial): Necitumumab + Gemcitabine-Cisplatin vs Gemcitabine-Cisplatin in 1st-Line Squamous NSCLC. *J Thorac Oncol* 2015; **10**: S797.

89 Herbst R, Redman M, Kim ES, *et al.* 3612: A randomized, phase III study comparing carboplatin/paclitaxel or carboplatin/paclitaxel/bevacizumab with or without concurrent cetuximab in patients with advanced non-small cell lung cancer (NSCLC): SWOG S0819. In: WCLC 2015. .

90 Carboplatin and Paclitaxel With or Without Bevacizumab and/or Cetuximab in Treating Patients With Stage IV or Recurrent Non-Small Cell Lung Cancer. ClinicalTrials.gov.gov. https://clinicaltrials.gov/ct2/show/NCT00946712 (accessed Nov 23, 2015).

91 Herbst R, Redman M, Kim ES, *et al.* PLEN04.01 A Randomized, Phase III Study Comparing Carboplatin/Paclitaxel or Carboplatin/Paclitaxel/Bevacizumab with or without Concurrent Cetuximab in Patients with Advanced Non-Small Cell Lung Cancer (NSCLC): SWOG S0819. *J Thorac Oncol* 2015; **Vol 10**: S795.

92 Takeda K, Hida T, Sato T, *et al.* Randomized phase III trial of platinum-doublet chemotherapy followed by gefitinib compared with continued platinum-doublet chemotherapy in Japanese patients with advanced non-small-cell lung cancer: results of a west Japan thoracic oncology group trial (W. *J Clin Oncol* 2010; **28**: 753–60.

93 Ahn M-J, Yang JC-H, Liang J, *et al.* Randomized phase II trial of first-line treatment with pemetrexed-cisplatin, followed sequentially by gefitinib or pemetrexed, in East Asian, never-smoker patients with advanced non-small cell lung cancer. *Lung Cancer* 2012; **77**: 346–52.

94 Karayama M, Inui N, Fujisawa T, *et al.* Maintenance therapy with pemetrexed and bevacizumab versus pemetrexed monotherapy after induction therapy with carboplatin , pemetrexed , and bevacizumab in patients with advanced non-squamous non small cell lung. *Eur J Cancer* 2016; **58**: 30–7.
